# Telomere Length and COVID-19 Outcomes: A Two-Sample Bidirectional Mendelian Randomization Study

**DOI:** 10.3389/fgene.2022.805903

**Published:** 2022-05-23

**Authors:** Li Jiang, Bei-sha Tang, Ji-feng Guo, Jin-chen Li

**Affiliations:** ^1^ National Clinical Research Center for Geriatric Disorders, Xiangya Hospital, Central South University, Changsha, China; ^2^ Center for Medical Genetics & Hunan Key Laboratory of Medical Genetics, School of Life Sciences, Central South University, Changsha, China; ^3^ Department of Neurology, Xiangya Hospital, Central South University, Changsha, China

**Keywords:** COVID-19, telomere length, aging, mendelian randomization, causality, genetic association

## Abstract

Observational studies have found a relationship between directly measured short leukocyte telomere length (LTL) and severe coronavirus disease 19 (COVID-19). We investigated the causal association between genetically predicted LTL and COVID-19 susceptibility or severity. A previous genome-wide association study (GWAS) of 78,592 European-ancestry participants identified single nucleotidepolymorphisms (SNPs) that can be utilized to genetically predict LTL. Summary-level data for COVID-19 outcomes were analyzed from the COVID-19 Host Genetics Initiative. A two-sample bidirectional Mendelian randomization (MR) study was designed to evaluate these causal relationships. Using an inverse-weighted MR analysis and population-based controls, genetically predicted LTL did not reveal any significant association with COVID-19 susceptibility (odds ratio (OR): 0.94; 95% CI: 0.85–1.04; *p* = 0.202) or severity (OR: 0.85; 95% CI: 0.70–1.03; *p* = 0.099). Similar results were found for five other definitions of cases/controls and/or COVID-19 outcomes. Six additional MR methods and sensitivity analyses were conducted after removing variants with potential horizontal pleiotropy and including variants at a liberal significance level, which produced similar results. Using SNPs identified for the prediction of LTL from another GWAS study, we found a non-significant association for COVID-19 susceptibility or severity with narrower CIs toward the null hypothesis. No proof of genetically predicted COVID-19 phenotypes remained causally associated with genetically predicted LTL, and the null association was consistent with a lack of significant genetic correlation. Genetic evidence does not support shorter LTL as a causal risk factor for COVID-19 susceptibility or severity.

## 1 Introduction

Telomere length (TL) has long been considered a candidate biomarker for cellular aging. Telomeres shorten progressively with age, resulting in DNA damage and the impairment of genome integrity with subsequent loss of replication ability, apoptosis, or cellular senescence when reaching a critical short length (Allsopp et al., 1992).

An increasing number of observational studies with different sample sizes and from multiple countries have found an association between shorter leukocyte TL (LTL) and increased risk of severe coronavirus disease 19 (COVID-19) (Froidure et al., 2020; Benetos et al., 2021; Sanchez-Vazquez et al., 2021; Wang et al., 2021). Although the associations between shorter LTL and risk of SARS-CoV-2 infection are yet to be investigated in observational studies, some evidence suggests that telomere-length-controlled replicative lifespan may play a role in the age-associated decline of T-cell functions (Son et al., 2000), a phenomenon denominated as immunosenescence. This phenomenon may increase susceptibility to infections (Cunha et al., 2020). COVID-19 has recently been considered an emergent aging-associated disease based on the evidence that the case-fatality rate of COVID-19 increases exponentially with age, and aging-associated chronic diseases are common poor prognostic factors in COVID-19 patients (Santesmasses et al., 2020). This causal relationship requires further investigation to determine whether LTL is a predictive biomarker of COVID-19 susceptibility or severity. Specifically, therapeutic agents targeting the telomere/telomerase system will be beneficial only if the alterations to the LTL are causal or contributory to the disease rather than a side-effect. Mendelian randomization (MR) is widely used to examine the potential causal relationship between exposure and disease outcome. MR studies use genetic variants as proxies for the exposure, which are independent of factors that may disrupt observational studies, thereby avoiding confounding bias and reverse causality. This study used a two-sample bidirectional MR design to estimate the causality of the relationship between genetically predicted LTL and COVID-19 susceptibility or severity, based on relevant GWAS summary statistics.

## 2 Materials and Methods

### 2.1 Leukocyte Telomere Length as Exposure

The single nucleotide polymorphisms (SNPs) that can be utilized to genetically predict LTL were obtained from a recent large-scale GWAS meta-analysis of up to 78,592 European-ancestry participants (Li et al., 2020) (Supplementary Tables S1, S2). The mean age was approximately 50.3 years (range, 24.3–73.4), with a similar proportion of men and women (55.5% women) (Li et al., 2020). LTL was measured using a quantitative polymerase chain reaction (qPCR)-based technique and was expressed as a T/S ratio. LTL within each cohort was standardized using a Z-transformation approach (Li et al., 2020). Age, sex, and study-specific covariates including batch, center, and genetic principle components, were adjusted in these studies (Li et al., 2020). We selected 20 genetic variants at 17 loci that were identified by Li et al. as strongly associated with directly measured LTL (*p* value <5 × 10^–8^) (Li et al., 2020) (Supplementary Table S3). To eliminate the possibility of remaining linkage disequilibrium (LD) between SNPs, we performed LD clumping to exclude SNPs that have an r^2^ > 0.05 with another variant within 1 Mb distance, which was based on 1,000 Genomes Data from non-Finnish European ancestry by LDlink tool (Machiela and Chanock, 2015). The SNP rs7705526 showed weak LD with rs2853677 (r^2^ = 0.16). Given that these SNPs were strongly associated with LTL (F statistics > 100, Supplementary Table S3) (Burgess and Thompson, 2011), they were retained for subsequent MR analysis. We searched for proxy SNPs (r^2^ > 0.7) using the LDlink tool (Machiela and Chanock, 2015) when the SNPs were not found in the GWAS dataset of outcome or with palindromic alleles of intermediate frequency (between 42 and 58%), based on the 1000 Genomes Project non-Finnish European dataset.

### 2.2 Outcome Data

COVID-19 phenotypic summary statistics (five round: A2, B1, B2, and C2; four round: A1, C1, and D1) were extracted from a GWAS meta-analysis performed by COVID-19 Host Genetics Initiative (HGI) (Initiative, 2020). Details of the phenotype definitions for the analyses (A1, A2, B1, B2, C1, C2, and D1) were described in Supplementary Tables S1, S7. Our analysis was limited to individuals of European ancestry excluding the 23andMe cohort. For the two primary analyses, COVID-19 susceptibility and severity were considered based on the largest number of cases and controls from general population. Susceptibility was defined by a positive test for current (determined by reverse transcription qPCR [RT-qPCR]) or past (serology) SARS-CoV-2 infection, or medical chart review, or ICD codes for the clinical diagnosis, or self-reporting of a positive test for SARS-CoV-2 infection (N = 38,984) versus population controls (N = 1,644,784) (C2). Severity was defined according to hospitalization of COVID-19 patients due to coronavirus symptoms, who were diagnosed by RT-qPCR, serology tests, or clinical diagnosis (N = 9,986) versus population controls (N = 1,877,672) (B2). Controls were not chosen according to any specific characteristics, testing status, or testing results, therefore they can be used to represent the general population to avoid selection, and collider bias. To determine the robustness of the results from our primary analyses, another five outcomes (C1 and D1 for susceptibility; B1, A1, and A2 for severity) based on different case and/or control definitions were used for the MR secondary analysis. Details from the COVID-19 HGI, such as types of studies in the research strategy, participating cohorts, case ascertainment, control selection, and the basic characteristics of the population in different COVID-19 phenotypes can be found in Supplementary Tables S4–S7.

### 2.3 MR Analysis

Prior to the analysis, these statistics were harmonized so that the effect size for the exposure and outcome corresponded to the same effect alleles. For the primary MR analysis, the inverse-variance weighted (IVW) method was conducted using a fixed-effects model (Burgess et al., 2015). The estimated MR association between genetically predicted LTL and COVID-19 susceptibility or severity (20 SNP set) was expressed as odds ratios (ORs) of COVID-19 outcomes per-standard deviation (SD) increase in genetically predicted LTL. To adjust for multiple testing, we used a significance threshold of 0.05/7 = 0.0071 by dividing by the number of outcomes based on the Bonferroni correction.

**FIGURE 1:**
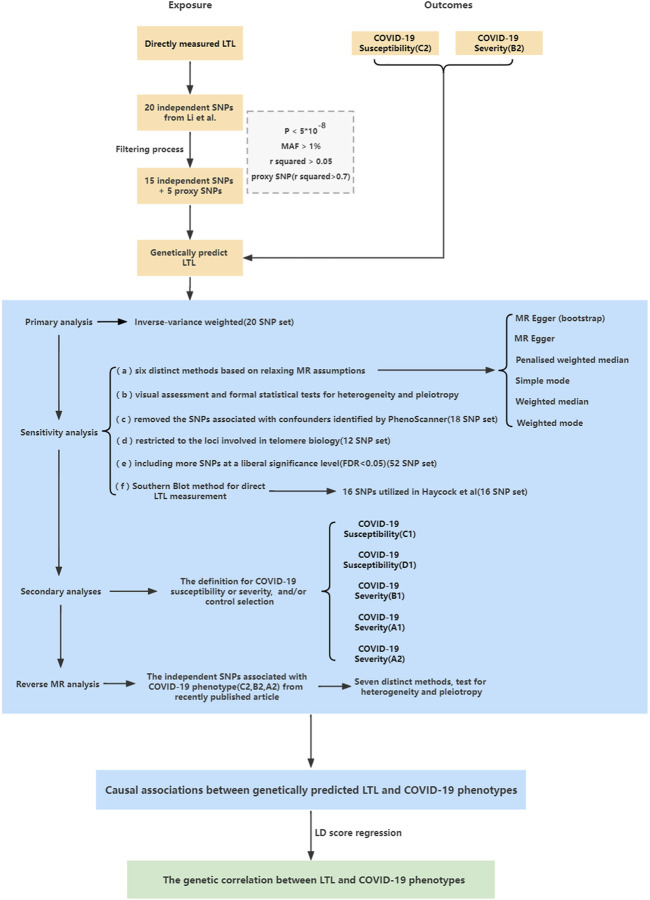
Study design overview.

To improve the reliability of the primary analysis, sensitivity analyses incorporating different approaches were designed and performed to detect potentially horizontal pleiotropic SNPs and to account for other potential biases. The design strategy is illustrated in Figure 1. 1) Six other MR models were used for MR estimates (Slob and Burgess, 2020). The boot-strap MR-Egger method was used in case of possible instability in MR-Egger estimates. The four remaining methods give more reliable results in the presence of horizontal pleiotropy, although at the cost of reduced statistical power; 2) To detect heterogeneity and possible horizontal pleiotropy, Cochran’s Q statistic, the intercept test, leave-one-out analysis, MR pleiotropy residual sum and outlier analysis (MR-PRESSO) (Verbanck et al., 2018) and funnel plot analysis were performed. 3) PhenoScanner (Kamat et al., 2019) resulted in the removal of 2 SNPs from the original 20 SNPs of Li et al. (18 SNP set) (Supplementary Table S3), because the 2 SNPs were previously reported associations with three plausible confounders (BMI, smoking, and alcoholic drinks) at *p* < 5 × 10^–8^, which have been reported to impact the risk of shorter LTL and COVID-19 severity (Valdes et al., 2005; Zhou et al., 2020; Maugeri et al., 2021). 4) We restricted our analysis to a subset of the 20 SNPs from Li et al. used in primary analyses (12 SNP set) encoding components of the SHELTERIN complex, regulating telomere structure, or regulating the formation and activity of telomerase (Li et al., 2020) (Supplementary Table S3); 5) To avoid the possibility of insufficiently powered instruments, we included more instruments up to 52 SNPs associated with LTL (Li et al., 2020) (52 SNP set) (Supplementary Table S3) at a liberal significance level (ranging from *p* = 5 × 10^–8^ to *p* = 1.03 × 10^–5^, equivalent to a false discovery rate of <0.05). 6) Given that Southern blot analysis is the gold standard for measuring LTL, although T/S ratios from qPCR and mean terminal restriction fragments from Southern blots were highly correlated, they did not yield identical results. To avoid bias from different methods for direct LTL measurement, another set of 16 SNPs was selected to genetically predict LTL as utilized in the Haycock et al. study (Haycock et al., 2017) (16 SNP set) (Supplementary Table S3). Briefly, the SNPs were selected from the GWAS catalog based on a reported *p* value <5 × 10^–8^, including top hits from two studies (Mangino et al., 2012; Codd et al., 2013), as well as other GWAS studies based on publicly available summary statistics from the GWAS catalog. The summary statistics of these SNPs were obtained by Haycock et al. from Mangino et al., which measured LTL using Southern blot analysis (Mangino et al., 2012). These details are presented in Supplementary Tables S1, S2.

Reverse MR analyses were conducted to explore the causal role of genetically predicted COVID-19 outcomes in LTL. Five SNPs for COVID-19 susceptibility (C2), and six and five SNPs for COVID-19 severity (B2 and A2) at *p* < 5 × 10^–8^ were used which were identified from a recent study (Supplementary Tables S8–S10) (Initiative, 2021), and the GWAS data of directly measured LTL were from Codd et al. (Codd et al., 2013) due to the full GWAS summary statistics from Li et al. unavailability. The reference and effect alleles were then harmonized for these SNPs according to the LTL and COVID-19 GWAS. The TwoSampleMR package was used in R for all MR analyses (Hemani et al., 2018).

### 2.4 Genetic Correlation

We estimated genetic correlations between genetically predicted LTL and COVID-19 outcomes (C2, B2, and A2) using LD score regression (LDSC) (Bulik-Sullivan et al., 2015) based on the 1,000 Genome Project Phase 3 (Auton et al., 2015) for European-ancestry population as a reference panel.

### 2.5 Ethics

The present study only used summary-level statistics from publicly available datasets. Data were not collected at the individual level. Therefore, ethical approval was not obtained.

## 3 Results

The 20 independent SNPs considered strong instruments (F-statistics ranging from 30.991 to 233.613) (Burgess and Thompson, 2011) explained 1.773% of the variance in LTL. Five of the 20 SNPs were absent in the COVID-19 GWAS and were replaced by proxies in high LD (r^2^ > 0.7) (Supplementary Tables S3, S11).

**FIGURE 2:**
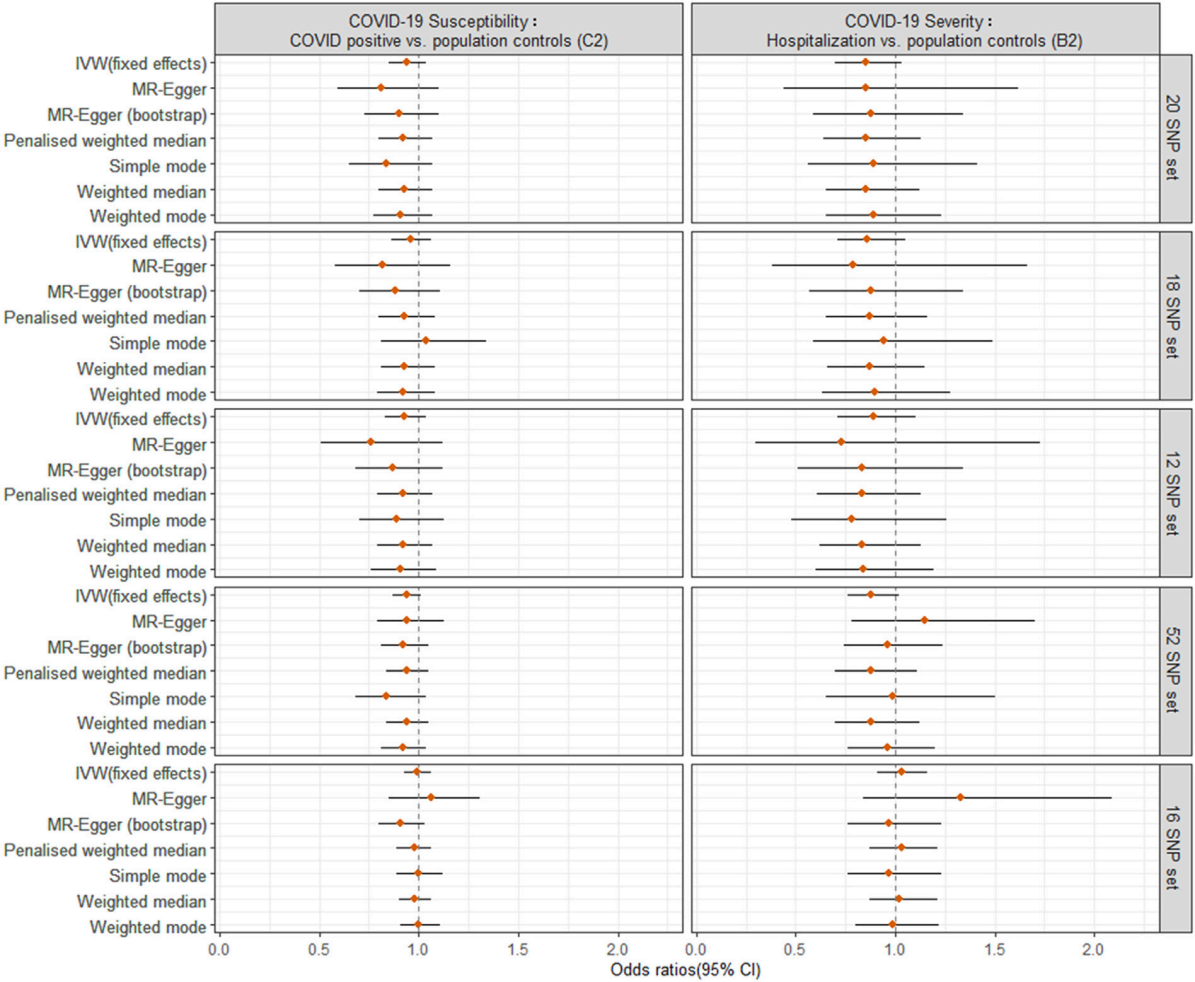
Odds ratio (OR) point estimates and 95% confidence intervals (CI) for the effect of a 1-SD increase in genetically predicted LTL from different sets of SNPs on COVID-19 susceptibility (C2 phenotype, left column) or severity (B2 phenotype, right column). For each set of SNPs (Supplementary Table S3), OR and CI are plotted for the inverse-variance weighted (IVW) fixed effects model from primary MR analyses and six additional methods from sensitivity analyses.

For the primary MR analysis utilizing the largest possible population-based controls (C2, B2), we found no significant effect of a SD increase in genetically predicted LTL on the odds of COVID-19 susceptibility (OR = 0.94; 95% CI: 0.85, 1.04; *p* = 0.202) or COVID-19 severity (OR = 0.85; 95% CI: 0.70, 1.03; *p* = 0.099) using IVW meta-analysis under a fixed-effects model. The results were not influenced by heterogeneity (IVW Cochran’s Q *p* > 0.05), directional horizontal pleiotropy (MR-Egger Intercept *p* > 0.05), or significant outliers (MR-PRESSO Global Test *p* > 0.05) (Figure 2, Supplementary Table S12).

For sensitivity analyses, the six meta-analyses provided similar point estimates, suggesting the robustness of our findings. Furthermore, we repeated the IVW analysis excluding the SNPs (rs2736176 and rs34978822) that were associated with hypertension and cerebral infarction (*p* < 5 × 10^–8^) using PhenoScanner (18 SNP set) and after including SNPs involved in telomere biology (12 SNP set), showing very similar results to the IVW estimate (Figure 2, Supplementary Table S12). To increase the power for MR estimation, we included 52 SNPs with a false discovery rate of <0.05, as previously reported (Li et al., 2020) (Supplementary Table S3). This could explain the 2.647% variance of LTL. The MR estimates for COVID-19 outcomes did not alter the inference of the primary results (*p* > 0.05) (52 SNP set). Considering the potential bias from different methods for direct LTL measurement, we used another 16 SNP instruments (explaining 3.566% variance of LTL measured by Southern blot analysis), and found the association between genetically predicted LTL and COVID-19 susceptibility (IVW: OR = 0.99, 95% CI: 0.93 to 1.06, *p* = 0.874) or severity (IVW: OR = 1.03, 95% CI: 0.91 to 1.16, *p* = 0.685) (16 SNP set). This produced similar null results to the primary analysis, although observed associations were not robust underlying different MR pleiotropy assumptions. Notably, the 95% CI became tight around the null, adding to the plausibility of a null causal finding.

**FIGURE 3:**
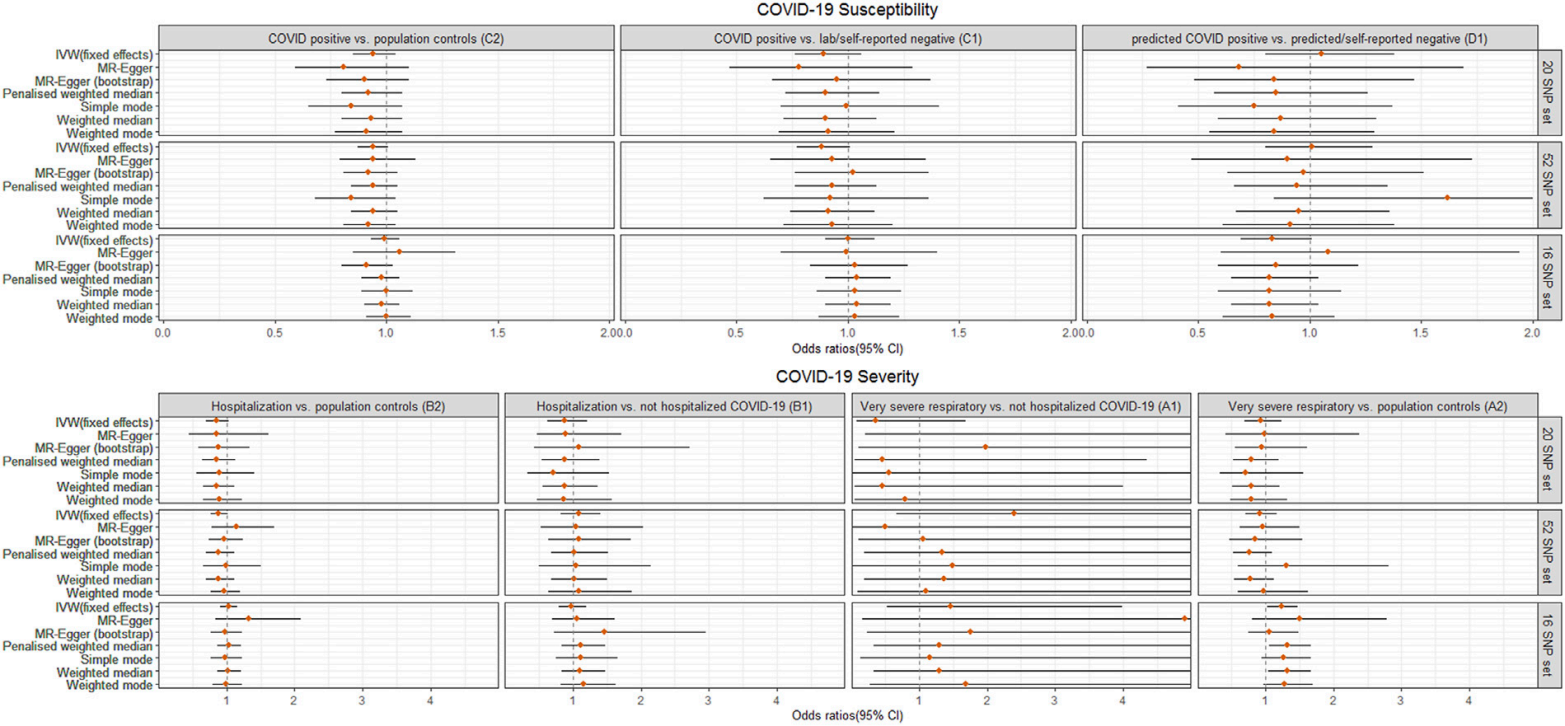
Odds ratio (OR) point estimates and 95% confidence intervals (CI) for the effect of a 1-SD increase in genetically predicted LTL from 3 sets of SNPs on primary (C2, B2) plus five additional phenotypes of COVID-19 susceptibility (C1, D1) or severity (B1, A1, A2), which vary by case definition and/or control selection (Supplementary Tables S1, S7). For each set of SNPs, OR and CI are plotted for the inverse-variance weighted (IVW) fixed effects model and six additional methods. Full results including OR, CI, and *p*-values are available in Supplementary Table S13.

For the different definitions of COVID-19 phenotypes and control selection in secondary analyses, genetically predicted LTL did not have a significant causal effect on COVID-19 susceptibility (C1, D1), severe (B1) or very severe COVID-19 (A1) outcomes, which was in line with the findings from the primary analysis (C2 and B2). Interestingly, we noted that genetically predicted longer LTL using 16 SNPs showed a nominally significant directionally consistent causal association with increased risk of very severe COVID-19 phenotype (A2: death OR respiratory support) using IVW, penalized weighted median and weighted median (Figure 3, Supplementary Table S13). However, this was not maintained over the Bonferroni-corrected significance threshold. The possible horizontal pleiotropy assessment was visualized for seven COVID-19 outcomes among the different SNP instrument sets in Supplementary Figures S1–S11 and Supplementary Tables S12, S13.

**FIGURE 4:**
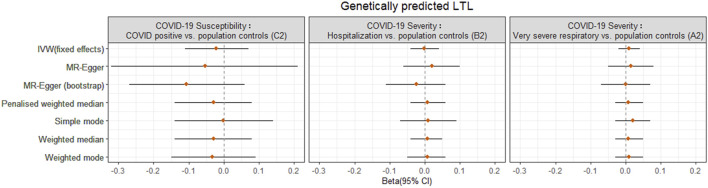
Odds ratio (OR) point estimates and 95% confidence intervals (CI) from the reverse MR analyses for the effect of COVID-19 phenotypes (susceptibility C2, severity B2, A2) on genetically predicted LTL from Codd et al. OR and CI are plotted for inverse-variance weighted (IVW) fixed effects model and six additional methods. Full results including OR, CI, and *p*-values are available in Supplementary Table S14.

For reverse MR analysis, there was no significant association between genetically predicted COVID-19 severity (B2, A2) or susceptibility (C2) and LTL across different MR methods, without evidence of heterogeneity and pleiotropy (Figure 4, Supplementary Table S14; Supplementary Figures S12–S14).

Finally, we failed to find any genetic correlation between LTL and COVID-19 phenotypes (*p* > 0.05) (Supplementary Table S15). Furthermore, the LDSC intercepts approached zero, demonstrating that the MR study had no substantial sample overlap between GWASs.

## 4 Discussion

Our study describes the first use of a two-sample bidirectional MR approach and a genome-wide genetic correlation analysis based on the summary-level data of two large GWASs. No evidence was found to support a causal relationship between genetically predicted shorter LTL and the risk of COVID-19 susceptibility or severity, and there was no significant genetic correlation or reverse causality between genetically predicted COVID-19 and LTL. Our results also raise the possibility but do not confirm, that genetically predicted longer LTL might increase the risk of very severe COVID-19, although the association did not survive Bonferroni correction.

To date, several small case-control studies on LTL and COVID-19 severe outcomes have been reported (Froidure et al., 2020; Benetos et al., 2021; Sanchez-Vazquez et al., 2021; Wang et al., 2021). Nevertheless, when LTL was measured at the time of hospital admission following SARS-CoV-2 infection, white cell turnover in response to infection might have affected the measurements, and reverse causal effects might have invalidated the results. Recently, Wang and colleagues (Wang et al., 2021) conducted a cohort study based on UK Biobank participants comparing LTL before SARS-CoV-2 infection. This study assessed 914 patients with a poor prognosis of COVID-19 (cases) and 5,861 patients who tested positive for COVID-19 in the community but were not admitted to the hospital (controls). They found a significantly shorter LTL in cases than in controls after adjusting for age, sex, and ethnicity (Wang et al., 2021). Overall, evidence from traditional epidemiological studies has suggested that a shorter LTL is associated with a greater risk of COVID-19 severity.

There may be several explanations for the discrepancy between epidemiological observations and the non-significant causal relationship between genetically predicted LTL and COVID-19 severity in our study. On one hand, it is important to note that such observational studies, despite attempts to adjust for potential confounding factors, have the possibility of significant residual confounding factors due to uncontrolled or imperfectly measured covariates (Cui and Tian, 2021). In particular, care must be taken to control for potential factors that might influence TL, such as technical variations in TL measurement, psychological stress, income, marital status, lifestyle, and cell-type composition (Demanelis et al., 2021). In addition, the participants from the UK Biobank tended to be relatively healthy, with low levels of many risk factors and mortality rates compared to the general population (Batty et al., 2020), and the participants were not recruited via random sampling as in the Wang et al. study (Wang et al., 2021). Since selection bias is likely to exist in ambiguous sample selection, association effects are not reliable indicators. On the other hand, TL was measured at one-time point in the previous studies, which may not be representative of true TL over a long period (Luu et al., 2019). In the present study, we selected sets of SNPs strongly identified with directly measured LTL and then obtained COVID-19 phenotypes from two large-scale GWAS summary datasets to analyze whether a causal relationship exists using multiple two-sample MR methods. The MR design describes the causal link between lifelong exposure and disease outcomes, precluding confounding and reverse causation (Smith and Ebrahim, 2003; Smith and Ebrahim, 2004). Additionally, these two GWAS datasets were generated from European individuals, reducing the effect of population stratification. Thus, compared with traditional observational approaches, the MR approach could provide more reliable estimates of the causal relationship between genetically predicted LTL and COVID-19 severity. Importantly, this result is also supported by the findings of a one-sample MR analysis of the UK Biobank cohort (Wang et al., 2021).

However, we have to acknowledge that our study has some limitations. First, this MR analysis was restricted to individuals of European ancestry. Thus, our results should be applied cautiously to people originating outside of Europe. Second, MR analysis assumes a linear relationship, and the summary-level data limited our investigation of the potential nonlinear roles of LTL on COVID-19 phenotypes. Third, our power for the MR estimate is limited by a small fraction of the variation in LTL (1–4%) (Pierce et al., 2011), although the COVID-19 HGI offers very large sample sizes, allowing us to identify strong-to-moderate associations (Supplementary Table S16). Fourthly, identifying cases during the COVID-19 pandemic poses another challenge. Like other COVID-19 MR studies, the controls might include “cases” that weren't identified since the majority of the cases were asymptomatic or they were not tested due to social distancing measures. In addition, some cases in the cohort of COVID-19 susceptibility were selected by self-reporting. We were unable to investigate how the potential misclassification influence our findings because we only had access to the summary statistics. Fifthly, the genetic variants used for MR have been linked to mean LTL in general population samples. Genetic variants may not be optimal if COVID-19 severity is primarily correlated with the shortest TL (Blackburn et al., 2015), although both measures are likely to be correlated. Finally, the genetic variants studied could not be compared to the measured TL, despite the fact that genetic variants will be less prone to confounding bias and issues of reverse causation as compared with observational studies. We note that the associations between LTL and COVID-19 susceptibility have not been established in observational studies. Therefore, the associations between genetically predicted shorter LTL and COVID-19 susceptibility or severity identified in our study need to be further examined in more well-designed observational or prospective studies.

## 5 Conclusion

In summary, our findings do not support the causal role of genetically predicted shorter LTL in the increased risk of COVID-19 phenotypes. This MR study found no support for LTL as a biomarker for predicting COVID-19 outcomes in the COVID-19 HGI cohort. Further MR studies using individual-level data based on larger sample sizes are required to confirm these findings and to delineate any potential nonlinear associations. More studies will be able to perform stratified analyses by age or sex, which is necessary to understand the causal pathways underpinning this association.

## Data Availability

The original contributions presented in the study are included in the article/Supplementary Material, further inquiries can be directed to the corresponding authors.
