# Supplementary material for: Telomere Length and COVID-19 Outcomes: A Two-Sample Bidirectional Mendelian Randomization Study
Source: Front Genet. 2022 May 23;13:805903. doi: 10.3389/fgene.2022.805903 (PMC9168682; doi:10.3389/fgene.2022.805903)
Supplement: Supplementary file 1 [file DataSheet1.docx]

| **Supplementary Table 1 Sources of data for the analysis** | | | | | | |
| --- | --- | --- | --- | --- | --- | --- |
| Phenotype | Cohort | | Participants | | | |
| Leukocyte telomere length | Mangino et al.2012  (MR sensitivity analyses) | | A meta-analysis of GWAS of LTL in 9,190 individuals of European ancestry from six collaborating studies:  **Mean Age:** 54, range from 35 to 75 years in individual cohort  **Sex:** 67% female  **Mean LTL:** 6.84(kb), range from 6.33 to 7.22 kb  **Measurement:** Southern blot analysis of the terminal restriction fragments(coefficients of variation ranged from 0.9 to 2.4% in replicate samples) and is expressed as the mean length of terminal restriction fragments  **Number of GWAS SNPs:** 2.5 million  **Imputation panel:** HapMap CEU using release 22 build 36  **Adjustments:** age, sex, BMI and smoking status (pack years) | | | |
|  | Codd et al.2013  (Reverse MR analyses) | | A meta-analysis of GWAS of LTL in 37,684 individuals of European ancestry from 15 cohorts:  **Mean Age:** 48.8, range from 18.7 to 71.7 in individual cohort  **Sex:** 58% female  **Mean LTL:** 1.80(T/S), range from 0.004 to 3.71  **Measurement:** Quantitative PCR-based technique and is expressed as a T/S ratio (coefficients of variation in individual cohorts ranged from 2.7 to 3.9%) and standardised LTL within each cohort using a Z-transformation approach  **Imputation panel:** HapMapII CEU build 36  **Number of GWAS SNPs:** 2,362,330  **Adjustments:** age, sex, and any study-specific covariates | | | |
|  | Li et al. 2020  (MR primary analyses) | | A genome-wide association (GWA) meta-analysis of 78,592 individuals of European ancestry from ENGAGE, EPIC-InterAct and EPIC-CVD studies:  **Mean Age:** 50.3, range from 24.3 to 73.4  **Sex:** 55.5% female  **Mean LTL:** 1.51(T/S) , range from 0.88 to 3.71  **Measurement:** Quantitative PCR-based technique and is expressed as a T/S ratio(coefficients of variation in individual cohorts ranged from 2.7 to 24.8%) and standardised LTL within each cohort using a Z-transformation approach  **Imputation panel:** 1000 Genomes EUR and Haplotype Reference Consortium build 37  **Number of GWAS SNPs:** not availiable  **Adjustments:** age, sex, and any study-specific covariates, including batch, center, and genetic principal components | | | |
| COVID-19 Susceptibility | Susceptibility(C1) | | Meta-analysis of 23 GWASs performed in individuals of European ancestry:  **Cases:** 24,057 individuals with COVID-19 by laboratory confirmation,chart review,or self-report  **Controls:** 218,062 individuals with COVID-19 negative by laboratory confirmation,chart review,or self-report  **Mean Age:** 51.3 for cases, range from 39.5 to 69.52 in individual cohort; 51.8 for controls, range from 39.5 to 67.92 in individual cohort  **Imputation panel:**HRC, TopMed, 1000Genomes version 3 phase 5, UK10K, whole genome sequencing of 49,708 Icelanders and Estonian population specific imputation reference  **Number of GWAS SNPs:** 13,060,710  **Adjustments:** age, age2, sex, age*sex, and 20 first principal compoments | | | |
|  | Susceptibility(C2) | | Meta-analysis of 35 GWASs performed in individuals of European ancestry:  **Cases:** 38,984 individuals with COVID-19 by laboratory confirmation,chart review,or self-report  **Controls:** 1,644,784 population controls  **Mean Age:** 54.6 for cases, range from 35.6 to 69.52 in individual cohort; 51.4 for controls range from 36.3 to 67.98 in individual cohort  **Imputation panel:** HRC, TopMed, 1000Genomes version 3, UK10K, whole genome sequencing of 49,708 Icelanders and Estonian population specific imputation reference  **Number of GWAS SNPs:** 8,738,878  **Adjustments:** age, age2, sex, age*sex, and 20 first principal compoments | | | |
|  | Susceptibility(D1) | | Meta-analysis of 6 GWASs performed in individuals of European ancestry:  **Cases:** 3,204 self-report COVID-19  **Controls:** 35,728 samples with minumum possible value from the predictive model AND not self-reported COVID-19 positive  **Mean Age:** 50.7 for cases, range from 41 to 58 in individual cohort; 50.7 for controls range from 41 to 58 in individual cohort  **Imputation panel:** HRC, TopMed, and 1000Genomes version 3  **Number of GWAS SNPs:** 11,480,752  **Adjustments:** age, age2, sex, age*sex, and 20 first principal compoments | | | |
| COVID-19 Severity | Hospitalized(B1) | | Meta-analysis of 12 GWASs performed in individuals of European ancestry:  **Cases:** 4,829 hospitalized individuals with COVID-19  **Controls:** 11,816 not hospitalized individuals with COVID-19  **Mean Age:** 57.4 for cases, range from 42 to 69.52 in individual cohort; 52.7 for controls range from 42 to 67.92 in individual cohort  **Imputation panel:** HRC, TopMed, 1000Genomes version 3 phase 5, UK10K, whole genome sequencing of 49,708 Icelanders and Estonian population specific imputation reference  **Number of GWAS SNPs:** 8,375,578  **Adjustments:** age, age2, sex, age*sex, and 20 first principal compoments | | | |
|  | Hospitalized(B2) | | Meta-analysis of 22 GWASs performed in individuals of European ancestry:  **Cases:** 9,986 hospitalized individuals with COVID-19  **Controls:** 1,877,672 population controls  **Mean Age:** 58.3 for cases, range from 42 to 72.29 in individual cohort; 55.1 for controls range from 47.92 to 67.98 in individual cohort  **Imputation panel:** HRC, TopMed, 1000Genomes version 3, UK10K, whole genome sequencing of 49,708 Icelanders and Estonian population specific imputation reference  **Number of GWAS SNPs:** 8,152,415  **Adjustments:** age, age2, sex, age*sex, and 20 first principal compoments | | | |
|  | Severe disease(A1) | | Meta-analysis of 4 GWASs performed in individuals of European ancestry:  **Cases:** 269 hospitalized individuals with COVID-19 AND (death OR respiratory support)  **Controls:** 688 not hospitalized individuals within 21 days after testing positive for COVID-19  **Mean Age:** 60.5 for cases, range from 48.4 to 72.29 in individual cohort; 58.0 for controls range from 47.92 to 67.55 in individual cohort  **Imputation panel:** HRC, TopMed, and 1000Genomes version 3  **Number of GWAS SNPs:** 9,304,135  **Adjustments:** age, age2, sex, age*sex, and 20 first principal compoments | | | |
|  | Severe disease(A2) | | Meta-analysis of 15 GWASs performed in individuals of European ancestry:  **Cases:** 5,101 hospitalized individuals with COVID-19 AND (death OR respiratory support)  **Controls:** 1,383,241 population controls  **Mean Age:** 61.9 for cases, range from 51 to 72.29 in individual cohort; 54.0 for controls range from 44.05 to 67.98 in individual cohort  **Imputation panel:** HRC, TopMed, and 1000Genomes version 3  **Number of GWAS SNPs:** 9,856,861  **Adjustments:** age, age2, sex, age*sex, and 20 first principal compoments | | | |
| **Supplementary Table 2 Cohort characteristics for the telomere length GWAS analysis** | | | | | |  |
| Cohort | | **Full name** | | **Ancestry** | **Sample** |  |
| **Mangino et al. 2012** | | | | | |  |
| FHS | | Framingham Heart Study | | EUR | 1146 |  |
| FamHS | | Family Heart Study | | EUR | 2508 |  |
| CHS | | Cardiovascular Health Study | | EUR | 1061 |  |
| BHS | | Bogalusa Heart Study | | EUR | 333 |  |
| HyperGEN | | Hypertension Genetic Epidemiology | | EUR | 920 |  |
| TwinsUK | | TwinsUK | | EUR | 3222 |  |
| **Codd et al. 2013** | | | | | |  |
| BHF-FHS | | British Heart Foundation Family Heart Study | | EUR | 1487 |  |
| EGCUT_370 | | Estonian Genome Center, University of Tartu_370 | | EUR | 2309 |  |
| EGCUT_OMNI | | Estonian Genome Center, University of Tartu _Omni genotyping Network Study | | EUR | 1251 |  |
| ERF | | Erasmus Rucphen Family | | EUR | 2581 |  |
| FINRISK | | Finrisk | | FIN | 520 |  |
| FTC/NAG-FIN | | Nicotine Addiction Genetics – Finland study | | FIN | 1054 |  |
| HBCS | | Helsinki Birth Cohort Study | | FIN | 1582 |  |
| KORA F3 | | Cooperative Health Research in the Region Augsburg F3 | | EUR | 1636 |  |
| KORA F4 | | Cooperative Health Research in the Region Augsburg F4 | | EUR | 1801 |  |
| LLS | | Leiden Longevity Study | | EUR | 2266 |  |
| NFBC1966 | | Northern Finland Birth Cohort 1966 | | FIN | 5146 |  |
| NTRMRG3 | | Netherlands Twin Register_MRG3 | | EUR | 2532 |  |
| NTR_DETECT | | Netherlands Twin Register_ DETECT | | EUR | 158 |  |
| NTR_GODOT | | Netherlands Twin Register_ GODOT | | EUR | 1435 |  |
| PREVEND | | Prevention of REnal and Vascular ENdstage Disease | | EUR | 2926 |  |
| QIMR | | Queensland Institute of Medical Research | | EUR | 2371 |  |
| TWINGENE | | TwinGene | | EUR | 300 |  |
| TWINSUK | | TwinsUK | | EUR | 4899 |  |
| UKBS | | United Kingdom Blood Service | | EUR | 1430 |  |
| **Li et al. 2020** | | | | | |  |
| EPIC-InterAct T2D cases | | European Prospective Investigation into Cancer and Nutrition-InterAct T2D cases | | EUR | 8499 |  |
| EPIC-InterAct subcohort | | European Prospective Investigation into Cancer and Nutrition-InterAct subcohort | | EUR | 12242 |  |
| EPIC-CVD CHD cases | | European Prospective Investigation into Cancer and Nutrition-cardiovascular disease coronary heart disease cases | | EUR | 7713 |  |
| EPIC-CVD CRBV cases | | European Prospective Investigation into Cancer and Nutrition-cardiovascular disease cerebrovascular cases | | EUR | 3450 |  |
| EPIC-CVD controls | | European Prospective Investigation into Cancer and Nutrition-cardiovascular disease controls | | EUR | 752 |  |
| BHF-FHS | | British Heart Foundation Family Heart Study | | EUR | 1487 |  |
| EGCUT_370 | | Estonian Genome Center, University of Tartu_370 | | EUR | 2354 |  |
| EGCUT_OMNI | | Estonian Genome Center, University of Tartu _Omni genotyping Network Study | | EUR | 2234 |  |
| ERF | | Erasmus Rucphen Family | | EUR | 2836 |  |
| FINRISK | | Finrisk | | FIN | 502 |  |
| FTC/NAG-FIN | | Nicotine Addiction Genetics – Finland study | | FIN | 831 |  |
| GRAPHIC | | Graphic | | EUR | 1011 |  |
| GENMETS cases | | Genmets cases | | FIN | 807 |  |
| GENMETS controls | | Genmets controls | | FIN | 1205 |  |
| HBCS | | Helsinki Birth Cohort Study | | FIN | 1699 |  |
| KORA F3 | | Cooperative Health Research in the Region Augsburg F3 | | EUR | 3047 |  |
| KORA F4 | | Cooperative Health Research in the Region Augsburg F4 | | EUR | 2907 |  |
| LLS | | Leiden Longevity Study | | EUR | 2320 |  |
| NESDA | | Netherlands Study of Depression and Anxiety | | EUR | 2190 |  |
| NFBC1966 | | Northern Finland Birth Cohort 1966 | | FIN | 5146 |  |
| NTR | | Netherlands Twin Register | | EUR | 4977 |  |
| QIMR | | Queensland Institute of Medical Research | | EUR | 2438 |  |
| RSI | | Rotterdam Study-I | | EUR | 1800 |  |
| RSIII | | Rotterdam Study-III | | EUR | 372 |  |
| TWINGENE | | TwinGene | | EUR | 295 |  |
| TWINSUK | | TwinsUK | | EUR | 4899 |  |
| UKBS | | United Kingdom Blood Service | | EUR | 1422 |  |

| **Supplementary Table 3 SNPs to genetically predict telomere length for our forward MR analyses** | | | | | | | | | | | | | |
| --- | --- | --- | --- | --- | --- | --- | --- | --- | --- | --- | --- | --- | --- |
| SNP  (gene) | Chr | BP(hg19) | EA | OA | EAF | Beta | SE | P-value | R^2^ | F statistic | Proxy SNP | r^2^ | Study* |
| rs10936600  (TERC)*^#^ | 3 | 169514585 | T | A | 0.24 | -0.086 | 0.006 | 7.18E-51 | 0.00271 | 213.374 | NA | NA | Li et al. 2020 |
| rs7705526  (TERT)*^#^ | 5 | 1285974 | A | C | 0.33 | 0.082 | 0.006 | 5.34E-45 | 0.00296 | 233.613 | rs7726159 | 0.74 | Li et al. 2020 |
| rs2853677  (TERT)*^#^ | 5 | 1287194 | A | G | 0.59 | -0.064 | 0.005 | 3.35E-31 | 0.00197 | 155.089 | NA | NA | Li et al. 2020 |
| rs4691895  (NAF1)*^#^ | 4 | 164048199 | C | G | 0.78 | 0.058 | 0.006 | 1.58E-21 | 0.00113 | 89.094 | NA | NA | Li et al. 2020 |
| rs9419958  (STN1 (OBFC1))*^#^ | 10 | 105675946 | C | T | 0.86 | -0.064 | 0.007 | 5.05E-19 | 0.00097 | 76.016 | NA | NA | Li et al. 2020 |
| rs75691080  (RTEL1/STMN3)*^#^ | 20 | 62269750 | T | C | 0.09 | -0.067 | 0.009 | 5.99E-14 | 0.00075 | 58.661 | NA | NA | Li et al. 2020 |
| rs59294613  (POT1)*^#^ | 7 | 124554267 | A | C | 0.29 | -0.041 | 0.005 | 1.17E-13 | 0.00069 | 53.984 | NA | NA | Li et al. 2020 |
| rs8105767  (ZNF208)* | 19 | 22215441 | G | A | 0.29 | 0.039 | 0.005 | 5.42E-13 | 0.00063 | 49.559 | NA | NA | Li et al. 2020 |
| rs73624724  (RTEL1/ZBTB46)*^#^ | 20 | 62436398 | C | T | 0.13 | 0.051 | 0.007 | 6.33E-12 | 0.00058 | 45.345 | NA | NA | Li et al. 2020 |
| rs3219104  (PARP1)*^#^ | 1 | 226562621 | C | A | 0.83 | 0.042 | 0.006 | 9.60E-11 | 0.00049 | 38.643 | rs907191 | 1.00 | Li et al. 2020 |
| rs2736176  (PRRC2A)* | 6 | 31587561 | C | G | 0.31 | 0.034 | 0.005 | 3.53E-10 | 0.00051 | 40.177 | NA | NA | Li et al. 2020 |
| rs3785074  (TERF2)*^#^ | 16 | 69406986 | G | A | 0.26 | 0.035 | 0.006 | 4.64E-10 | 0.00048 | 37.510 | NA | NA | Li et al. 2020 |
| rs7194734  (MPHOSPH6)* | 16 | 82199980 | T | C | 0.78 | -0.037 | 0.006 | 6.94E-10 | 0.00047 | 36.628 | NA | NA | Li et al. 2020 |
| rs34978822  (RTEL1)*^#^ | 20 | 62291599 | G | C | 0.01 | -0.140 | 0.023 | 7.26E-10 | 0.00057 | 44.827 | NA | NA | Li et al. 2020 |
| rs34991172  (CARMIL1)* | 6 | 25480328 | G | T | 0.07 | -0.061 | 0.010 | 6.19E-09 | 0.00047 | 37.032 | NA | NA | Li et al. 2020 |
| rs228595  (ATM)*^#^ | 11 | 108105593 | A | G | 0.42 | -0.028 | 0.005 | 1.43E-08 | 0.00039 | 30.991 | NA | NA | Li et al. 2020 |
| rs2302588  (DCAF4)* | 14 | 73404752 | C | G | 0.10 | 0.048 | 0.008 | 1.68E-08 | 0.00041 | 32.150 | rs77694099 | 1.00 | Li et al. 2020 |
| rs13137667  (MOB1B )* | 4 | 71774347 | C | T | 0.96 | 0.077 | 0.014 | 2.43E-08 | 0.00046 | 36.102 | rs28668618 | 0.90 | Li et al. 2020 |
| rs55749605  (SENP7)* | 3 | 101232093 | A | C | 0.58 | -0.037 | 0.007 | 2.45E-08 | 0.00068 | 53.346 | rs13322987 | 1.00 | Li et al. 2020 |
| rs62053580  (RFWD3)* | 16 | 74680074 | G | C | 0.17 | -0.039 | 0.007 | 4.08E-08 | 0.00042 | 33.405 | NA | NA | Li et al. 2020 |
| rs932827  (ZBTB46) | 20 | 62380527 | T | C | 0.24 | -0.037 | 0.006 | 3.28E-10 | 0.00051 | 40.101 | NA | NA | Li et al. 2020 |
| rs754017156  (ACYP2) | 2 | 54482703 | D | GGGCCC | 0.17 | 0.047 | 0.009 | 7.52E-08 | 0.00061 | 47.969 | rs1872329 | 0.97 | Li et al. 2020 |
| rs12909131  (ATP8B4) | 15 | 50387678 | T | C | 0.23 | -0.031 | 0.006 | 1.15E-07 | 0.00034 | 26.730 | NA | NA | Li et al. 2020 |
| rs1744757  (MROH8 ) | 20 | 35734863 | T | C | 0.85 | 0.036 | 0.007 | 1.38E-07 | 0.00033 | 25.943 | rs6124509/rs2880234 | 1.00 | Li et al. 2020 |
| rs2124616  (TYMS) | 18 | 661917 | A | G | 0.14 | -0.037 | 0.007 | 1.72E-07 | 0.00034 | 26.730 | NA | NA | Li et al. 2020 |
| rs2613954  (RP11-572M11.4) | 3 | 112847045 | T | C | 0.89 | -0.038 | 0.008 | 1.10E-06 | 0.00029 | 22.798 | NA | NA | Li et al. 2020 |
| rs12065882  (MAGI3) | 1 | 114078755 | G | A | 0.21 | 0.030 | 0.006 | 1.36E-06 | 0.00029 | 22.798 | NA | NA | Li et al. 2020 |
| rs2386642  (ASB13) | 10 | 5702259 | A | G | 0.67 | -0.026 | 0.005 | 1.44E-06 | 0.00029 | 22.798 | NA | NA | Li et al. 2020 |
| rs56810761  (UNC80) | 2 | 210663697 | T | C | 0.27 | 0.027 | 0.006 | 1.45E-06 | 0.00030 | 23.584 | NA | NA | Li et al. 2020 |
| rs62365174  (TENT2) | 5 | 78925743 | G | A | 0.09 | -0.054 | 0.011 | 1.50E-06 | 0.00048 | 37.741 | NA | NA | Li et al. 2020 |
| rs112655343  (ATF7IP) | 12 | 14430807 | T | C | 0.10 | 0.043 | 0.009 | 2.22E-06 | 0.00033 | 25.943 | NA | NA | Li et al. 2020 |
| rs55710439  (ANKDD1A) | 15 | 65229816 | T | C | 0.01 | 0.105 | 0.022 | 2.65E-06 | 0.00031 | 24.370 | rs73473246 | 1.00 | Li et al. 2020 |
| rs60160057  (DCLK2) | 4 | 151000830 | A | G | 0.21 | -0.029 | 0.006 | 3.15E-06 | 0.00028 | 22.011 | NA | NA | Li et al. 2020 |
| rs117536281  (CDCA4) | 14 | 105494403 | G | A | 0.03 | 0.085 | 0.018 | 3.31E-06 | 0.00048 | 37.741 | NA | NA | Li et al. 2020 |
| rs59192843  (BBOF1) | 14 | 74514120 | G | T | 0.06 | 0.065 | 0.014 | 3.52E-06 | 0.00048 | 37.741 | rs199567548 | 0.90 | Li et al. 2020 |
| rs57415150  (CSMD1) | 8 | 2882469 | A | G | 0.04 | -0.058 | 0.013 | 3.68E-06 | 0.00027 | 21.225 | rs17079101 | 1.00 | Li et al. 2020 |
| rs6107615  (PROKR2) | 20 | 5310273 | C | T | 0.42 | -0.023 | 0.005 | 5.30E-06 | 0.00025 | 19.652 | NA | NA | Li et al. 2020 |
| rs117037102  (CEP295) | 11 | 93404608 | T | C | 0.02 | 0.098 | 0.022 | 6.81E-06 | 0.00034 | 26.730 | NA | NA | Li et al. 2020 |
| rs11665818  (IFNL2) | 19 | 39768216 | A | G | 0.19 | 0.028 | 0.006 | 7.04E-06 | 0.00024 | 18.866 | NA | NA | Li et al. 2020 |
| rs3213718  (CALM1) | 14 | 90869913 | T | C | 0.58 | 0.022 | 0.005 | 7.22E-06 | 0.00024 | 18.866 | NA | NA | Li et al. 2020 |
| rs143276018  (NMRK2) | 19 | 3939249 | C | T | 0.02 | -0.102 | 0.023 | 9.02E-06 | 0.00037 | 29.089 | NA | NA | Li et al. 2020 |
| rs201375979  (COX6C) | 8 | 100917632 | D | TA | 0.32 | 0.033 | 0.007 | 9.11E-06 | 0.00048 | 37.741 | rs7832556 | 1.00 | Li et al. 2020 |
| rs7311314  (SMUG1) | 12 | 54592103 | A | G | 0.32 | 0.024 | 0.005 | 9.50E-06 | 0.00025 | 19.652 | rs11170843 | 1.00 | Li et al. 2020 |
| rs35675808  (CD247) | 1 | 167399643 | G | C | 0.03 | 0.074 | 0.017 | 9.54E-06 | 0.00030 | 23.584 | NA | NA | Li et al. 2020 |
| rs7276273  (KRTAP10-4) | 21 | 45994841 | C | A | 0.01 | -0.150 | 0.033 | 6.90E-06 | 0.00033 | 25.943 | NA | NA | Li et al. 2020 |
| rs7510583  (KIAA1644) | 22 | 44698803 | G | A | 0.29 | 0.035 | 0.007 | 3.38E-06 | 0.00050 | 39.315 | NA | NA | Li et al. 2020 |
| rs11125529  (ACYP2) | 2 | 54475866 | A | C | 0.16 | 0.065 | 0.012 | 6.06E-03 | 0.00080 | 7.346 | rs62139258 | 1.00 | Haycock et al. 2017 |
| rs6772228  (PXK) | 3 | 58376019 | T | A | 0.87 | 0.041 | 0.014 | 4.97E-02 | 0.00200 | 17.291 | NA | NA | Haycock et al. 2017 |
| rs12696304  (TERC) | 3 | 169481271 | C | G | 0.74 | 0.090 | 0.011 | 5.41E-08 | 0.00319 | 28.834 | NA | NA | Haycock et al. 2017 |
| rs10936599  (TERC) | 3 | 169492101 | C | T | 0.76 | 0.100 | 0.011 | 1.76E-09 | 0.00319 | 29.404 | NA | NA | Haycock et al. 2017 |
| rs1317082  (TERC) | 3 | 169497585 | A | G | 0.71 | 0.097 | 0.011 | 4.57E-09 | 0.00319 | 29.359 | NA | NA | Haycock et al. 2017 |
| rs10936601  (TERC) | 3 | 169528449 | C | T | 0.74 | 0.087 | 0.011 | 8.64E-08 | 0.00319 | 29.276 | NA | NA | Haycock et al. 2017 |
| rs7675998  (NAF1) | 4 | 164007820 | G | A | 0.80 | 0.048 | 0.012 | 1.00E-02 | 0.00190 | 17.435 | rs4295216 | 1.00 | Haycock et al. 2017 |
| rs2736100  (TERT) | 5 | 1286516 | C | A | 0.52 | 0.085 | 0.013 | 2.14E-05 | 0.00310 | 17.893 | NA | NA | Haycock et al. 2017 |
| rs9419958  (OBFC1) | 10 | 105675946 | T | C | 0.13 | 0.129 | 0.013 | 5.26E-11 | 0.00171 | 15.738 | NA | NA | Haycock et al. 2017 |
| rs9420907  (OBFC1) | 10 | 105676465 | C | A | 0.14 | 0.142 | 0.014 | 1.14E-11 | 0.00171 | 15.738 | rs11591710 | 1.00 | Haycock et al. 2017 |
| rs4387287  (OBFC1) | 10 | 105677897 | A | C | 0.14 | 0.120 | 0.013 | 1.40E-09 | 0.00171 | 14.627 | NA | NA | Haycock et al. 2017 |
| rs3027234  (CTC1) | 17 | 8136092 | C | T | 0.83 | 0.103 | 0.012 | 2.75E-08 | 0.00292 | 26.667 | NA | NA | Haycock et al. 2017 |
| rs8105767  (ZNF208) | 19 | 22215441 | G | A | 0.25 | 0.064 | 0.011 | <0.001 | 0.00090 | 8.192 | NA | NA | Haycock et al. 2017 |
| rs412658  (ZNF676) | 19 | 22359440 | T | C | 0.35 | 0.086 | 0.010 | 1.83E-08 | 0.00484 | 44.521 | NA | NA | Haycock et al. 2017 |
| rs6028466  (DHX35) | 20 | 38129002 | A | G | 0.17 | 0.058 | 0.013 | 4.00E-03 | 0.00041 | 3.769 | NA | NA | Haycock et al. 2017 |
| rs755017  (ZBTB46) | 20 | 62421622 | G | A | 0.17 | 0.019 | 0.013 | 3.40E-01 | 0.00090 | 7.228 | rs2281929 | 1.00 | Haycock et al. 2017 |
| *The SNPs were included in 20 SNP set for the primary MR analysis  ^#^The SNPs were included in 12 SNP set for the MR sensitivity analysis  Chr: Chromosome; EA: Effect allele; OA: Other allele; EAF: Effect allele frequency; SE: Standard error | | | | | | | | | | | | | |

| **Supplementary Table 4 Susceptibility phenotype for COVID-19** | | | | | | |
| --- | --- | --- | --- | --- | --- | --- |
| Cohort | **Full name** | **Ancestry** | **Cases** | **Controls** | | **Design** |
| **C2_ALL_eur** | | | | | | |
| BQC19 | Biobanque Quebec COVID19 | EUR | 269 | | 368 | Prospective |
| BelCovid | Genetic modifiers for COVID-19 related illness | EUR | 485 | | 1477 | Prospective |
| BioVU | Vanderbilt Biobank | EUR | 141 | | 70615 | Retrospective |
| CCPM | The Colorado Center for Personalized Medicine | EUR | 332 | | 32375 | Retrospective |
| CU | Columbia University COVID19 Biobank | EUR | 508 | | 2149 | Retrospective, Prospective |
| DECODE | deCODE | EUR | 4256 | | 270934 | Retrospective |
| EstBB | Estonian Biobank | EUR | 1322 | | 193774 | Retrospective, Prospective |
| FinnGen | FinnGen | FIN | 810 | | 237901 | Retrospective |
| GCAT | Genomes for Life | EUR | 253 | | 4735 | Retrospective, Prospective |
| GENCOVID | GEN-COVID, reCOVID | EUR | 1220 | | 2443 | Retrospective, Prospective |
| GFG | Genes for Good | EUR | 147 | | 5442 | Retrospective |
| GHS_Freeze_145 | Geisinger Health System | EUR | 869 | | 112862 | Retrospective |
| Genotek | Genotek COVID-19 study | EUR | 676 | | 12317 | Prospective |
| INTERVAL | UK Blood Donors Cohort | EUR | 838 | | 40994 | Retrospective |
| LGDB | Latvia COVID-19 research platform | EUR | 275 | | 1313 | Prospective |
| Lifelines | Lifelines | EUR | 244 | | 26553 | Retrospective |
| MSHS_CGI | Mount Sinai Health System COVID-19 Genomics Initiative | EUR | 330 | | 1396 | Retrospective, Prospective |
| Stanford | Genomic epidemiology of SARS-Cov-2 and host genetics in Coronavirus Disease 2019 (COVID-19) | EUR | 169 | | 190 | Retrospective, Prospective |
| TOPMed_CHRIS10K | TOPMed CHRIS | EUR | 92 | | 2373 | Retrospective, Prospective |
| TOPMed_Gardena | TOPMed Gardena | EUR | 452 | | 458 | Retrospective |
| UCLA | UCLA Precision Health COVID-19 Biobank | EUR | 203 | | 17391 | Retrospective, Prospective |
| SPGRX | Determining the Molecular Pathways and Genetic Predisposition of the Acute Inflammatory Process Caused by SARS-CoV-2 | EUR | 362 | | 302 | Prospective |
| MVP | Million Veterans Program | EUR | 1520 | | 7600 | Retrospective |
| genomicsengland100kgp | Genomics England | EUR | 218 | | 62302 | NA |
| Helix | Helix Exome+ COVID-19 Phenotypes | EUR | 178 | | 5441 | Retrospective |
| MGI | Michigan Genomics Initiative | EUR | 122 | | 51458 | Retrospective, Prospective |
| NTR | Netherlands Twin Register | EUR | 145 | | 5252 | Retrospective, Prospective |
| PHBB | Partners Healthcare Biobank | EUR | 151 | | 29966 | Retrospective, Prospective |
| Ancestry | Ancestry | EUR | 2417 | | 14933 | Retrospective |
| idipaz24genetics | 24Genetics | EUR | 106 | | 75 | Retrospective, Prospective |
| Amsterdam_UMC_COVID_study_group | Amsterdam UMC COVID study group | EUR | 108 | | 1413 | Prospective |
| HOSTAGE | COVID19-Host(a)ge | EUR | 1610 | | 2205 | Retrospective |
| SweCovid | The genetic predisposition to severe COVID-19 | EUR | 77 | | 3748 | Prospective |
| genomicc | genomiCC | EUR | 1676 | | 8380 | Prospective |
| **C1_ALL** | | | | | | |
| UKBB | UK Biobank | AFR | 68 | | 268 | Retrospective |
| UKBB | UK Biobank | CSA | 66 | | 350 | Retrospective |
| UKBB | UK Biobank | EUR | 1310 | | 13646 | Retrospective |
| DECODE | deCODE | EUR | 1897 | | 29014 | Retrospective |
| EstBB | Estonian Biobank | EUR | 313 | | 12019 | Retrospective, Prospective |
| Genomicsengland100kgp | Genomics England | EUR | 218 | | 1617 | NA |
| GNH | Genes & Health | SAS | 114 | | 256 | Retrospective |
| Lifelines | Lifelines | EUR | 358 | | 1253 | Retrospective |
| MGI | Michigan Genomics Initiative | EUR | 122 | | 508 | Retrospective, Prospective |
| MVP | Million Veterans Program | AFR | 1217 | | 9204 | Retrospective |
| MVP | Million Veterans Program | EUR | 1520 | | 24135 | Retrospective |
| MVP | Million Veterans Program | HIS | 510 | | 3325 | Retrospective |
| NTR | Netherlands Twin Register | EUR | 145 | | 117 | Retrospective, Prospective |
| PHBB | Partners Healthcare Biobank | AFR | 60 | | 375 | Retrospective, Prospective |
| PHBB | Partners Healthcare Biobank | EUR | 151 | | 3118 | Retrospective, Prospective |
| PHBB | Partners Healthcare Biobank | HIS | 66 | | 276 | Retrospective, Prospective |
| PMBB | Penn Medicine Biobank | AFR | 166 | | 934 | Retrospective, Prospective |
| BQC19 | Biobanque Quebec COVID19 | EUR | 206 | | 327 | Prospective |
| INTERVAL | UK Blood Donors Cohort | EUR | 161 | | 1119 | Retrospective |
| Ancestry | Ancestry | EUR | 2417 | | 14933 | Retrospective |
| **D1_ALL** | | | | | | |
| GS | Generation Scotland | EUR | 132 | | 3610 | Retrospective |
| GeneRISK | Gene Risk | EUR | 154 | | 3451 | Retrospective, Prospective |
| Helix | Helix Exome+ COVID-19 Phenotypes | EUR | 605 | | 4778 | Retrospective |
| Lifelines | Lifelines | EUR | 1427 | | 16833 | Retrospective |
| NTR | Netherlands Twin Register | EUR | 603 | | 4669 | Retrospective, Prospective |
| RS | Rotterdam Study | EUR | 283 | | 2387 | Retrospective |

| **Supplementary Table 5 Hospitalized phenotype for COVID-19** | | | | | |
| --- | --- | --- | --- | --- | --- |
| Cohort | **Full name** | **Ancestry** | **Cases** | **Controls** | **Design** |
| **B2_ALL_eur** | | | | | |
| BQC19 | Biobanque Quebec COVID19 | EUR | 244 | 396 | Prospective |
| BelCovid | Genetic modifiers for COVID-19 related illness | EUR | 363 | 1477 | Prospective |
| CU | Columbia University COVID19 Biobank | EUR | 453 | 2149 | Retrospective, Prospective |
| EstBB | Estonian Biobank | EUR | 90 | 196339 | Retrospective, Prospective |
| FinnGen | FinnGen | FIN | 106 | 238605 | Retrospective |
| GENCOVID | GEN-COVID, reCOVID | EUR | 893 | 2443 | Retrospective, Prospective |
| GHS_Freeze_145 | Geisinger Health System | EUR | 180 | 112862 | Retrospective |
| LGDB | Latvia COVID-19 research platform | EUR | 57 | 1531 | Prospective |
| UCLA | UCLA Precision Health COVID-19 Biobank | EUR | 80 | 17514 | Retrospective, Prospective |
| UKBB | UK Biobank | EUR | 1670 | 328577 | Retrospective |
| idipaz24genetics | 24Genetics | EUR | 106 | 75 | Retrospective, Prospective |
| Amsterdam_UMC_COVID_study_group | Amsterdam UMC COVID study group | EUR | 108 | 1413 | Prospective |
| SPGRX | Determining the Molecular Pathways and Genetic Predisposition of the Acute Inflammatory Process Caused by SARS-CoV-2 | EUR | 311 | 302 | Prospective |
| DECODE | deCODE | EUR | 89 | 274322 | Retrospective |
| MVP | Million Veterans Program | EUR | 436 | 2180 | Retrospective |
| HOSTAGE | COVID19-Host(a)ge | EUR | 1610 | 2205 | Retrospective |
| BoSCO | Bonn Study of COVID19 genetics | EUR | 212 | 512 | Retrospective, Prospective |
| FHoGID | TOPMed CHRIS | EUR | 362 | 259 | Retrospective, Prospective |
| Ancestry | TOPMed Gardena | EUR | 250 | 1967 | Retrospective |
| SweCovid | The genetic predisposition to severe COVID-19 | EUR | 77 | 3748 | Prospective |
| genomicc | genomiCC | EUR | 1676 | 8380 | Prospective |
| **B1_ALL_eur** | | | | | |
| BelCovid | Genetic modifiers for COVID-19 related illness | EUR | 361 | 122 | Prospective |
| BoSCO | Bonn Study of COVID19 genetics | EUR | 212 | 512 | Retrospective, Prospective |
| EstBB | Estonian Biobank | EUR | 60 | 512 | Retrospective, Prospective |
| FHoGID | FHoGID | EUR | 362 | 259 | Prospective |
| FinnGen | FinnGen | FIN | 106 | 520 | Retrospective |
| GENCOVID | GEN-COVID, reCOVID | EUR | 892 | 249 | Retrospective, Prospective |
| UCLA | UCLA Precision Health COVID-19 Biobank | EUR | 80 | 123 | Retrospective, Prospective |
| UKBB | UK Biobank | EUR | 1670 | 4610 | Retrospective |
| SPGRX | Determining the Molecular Pathways and Genetic Predisposition of the Acute Inflammatory Process Caused by SARS-CoV-2 | EUR | 311 | 51 | Prospective |
| DECODE | deCODE | EUR | 89 | 1808 | Retrospective |
| MVP | Million Veterans Program | EUR | 436 | 1083 | Retrospective |
| Ancestry | Ancestry | EUR | 250 | 1967 | Retrospective |

| **Supplementary Table 6 Severe disease phenotype for COVID-19** | | | | | |
| --- | --- | --- | --- | --- | --- |
| Cohort | **Full name** | **Ancestry** | **Cases** | **Controls** | **Design** |
| **A2_ALL_eur** | | | | | |
| BQC19 | Biobanque Quebec COVID19 | EUR | 88 | 552 | Prospective |
| BelCovid | Genetic modifiers for COVID-19 related illness | EUR | 182 | 1477 | Prospective |
| CU | Columbia University COVID19 Biobank | EUR | 203 | 2149 | Retrospective, Prospective |
| FinnGen | FinnGen | FIN | 68 | 238643 | Retrospective |
| GENCOVID | GEN-COVID, reCOVID | EUR | 724 | 2443 | Retrospective, Prospective |
| GHS_Freeze_145 | Geisinger Health System | EUR | 53 | 112862 | Retrospective |
| SweCovid | The genetic predisposition to severe COVID-19 | EUR | 77 | 3748 | Prospective |
| UKBB | UK Biobank | EUR | 309 | 328577 | Retrospective |
| idipaz24genetics | 24Genetics | EUR | 59 | 75 | Retrospective, Prospective |
| Amsterdam_UMC_COVID_study_group | Amsterdam UMC COVID study group | EUR | 66 | 1413 | Prospective |
| SPGRX | Determining the Molecular Pathways and Genetic Predisposition of the Acute Inflammatory Process Caused by SARS-CoV-2 | EUR | 101 | 302 | Prospective |
| genomicc | genomiCC | EUR | 1676 | 8380 | Prospective |
| Italy_HOSTAGE | Italy COVID19-Host(a)ge | EUR | 698 | 1255 | Retrospective |
| Spain_HOSTAGE | Spain COVID19-Host(a)ge | EUR | 302 | 925 | Retrospective |
| **A1_ALL** | | | | | |
| BoSCO | Bonn Study of COVID19 genetics | EUR | 59 | 262 | Retrospective, Prospective |
| FinnGen | FinnGen | FIN | 54 | 224 | Retrospective |
| SPGRX | Determining the Molecular Pathways and Genetic Predisposition of the Acute Inflammatory Process Caused by SARS-CoV-2 | EUR | 101 | 51 | Prospective |
| BQC19 | Biobanque Quebec COVID19 | EUR | 55 | 151 | Prospective |

| **Supplementary Table 7 Definition of the COVID-19 phenotypes, as per extracted from COVID-19 Host Genetics Initiative (**[**https://www.covid19hg.org/**](https://www.covid19hg.org/)**)** | |
| --- | --- |
| Phenotype | Case ascertainment |
| COVID-19 Susceptibility (C1, C2) | Individuals with laboratory confirmation of SARS-CoV-2 infection (RNA and/or serology based) OR EHR/ICD coding/Physician Confirmed COVID-19 OR self-reported COVID-19 positive (e.g. by questionnaire) |
| COVID-19 predicted from self-reported symptoms (D1) | Samples with a value from the predictive model > -0.44 OR self-reported COVID-19 positive, the model from Menni et al. 2020, minus age and sex predictors, will be applied to self-report data. |
| Hospitalized lab confirmed COVID-19 (B1, B2) | Hospitalized laboratory confirmed SARS-CoV-2 infection (RNA and/or serology based), hospitalization due to corona-related symptoms. |
| Very severe respiratory confirmed COVID-19 (A1, A2) | Hospitalized laboratory confirmed SARS-CoV-2 infection (RNA and/or serology based), AND (death OR respiratory support (intubation, CPAP, BiPAP, CNP (continue external negative pressure), Optiflow/very high flow Positive End Expiratory Pressure Oxygen AND hospitalization with COVID-19 as primary reason for admission. |

| **Supplementary Table 8 SNPs from the Covid-Host Genetics Initiative to predict COVID-19 susceptibility (C2) for reverse MR analyses** | | | | | | | | | | | | | |
| --- | --- | --- | --- | --- | --- | --- | --- | --- | --- | --- | --- | --- | --- |
| SNP  (gene) | Chr | BP(hg19) | EA | OA | EAF | Beta | SE | P-value | R^2^ | F statistic | Proxy SNP | r^2^ | Study |
| rs10490770  (LZTFL1) | 3 | 45864732 | C | T | 0.08 | 0.159 | 0.014 | 2.14E-28 | 0.00372 | 6286.997 | NA | 1.00 | COVID-19 HGI. 2021 |
| rs11919389  (RPL24) | 3 | 101424458 | C | T | 0.36 | -0.067 | 0.009 | 5.44E-15 | 0.00207 | 3492.625 | NA | 1.00 | COVID-19 HGI. 2021 |
| rs529565  (ABO) | 9 | 136149500 | G | A | 0.65 | -0.106 | 0.009 | 4.40E-35 | 0.00511 | 8591.332 | rs687289 | 0.94 | COVID-19 HGI. 2021 |
| rs10774671  (OAS1) | 12 | 113357193 | A | G | 0.67 | 0.061 | 0.010 | 1.29E-10 | 0.00165 | 2780.708 | NA | 1.00 | COVID-19 HGI. 2021 |
| rs2109069  (DPP9) | 19 | 4719443 | A | G | 0.32 | 0.049 | 0.009 | 2.86E-08 | 0.00104 | 1736.505 | NA | 1.00 | COVID-19 HGI. 2021 |
| Chr: Chromosome; EA: Effect allele; OA: Other allele; EAF: Effect allele frequency; SE: Standard error | | | | | | | | | | | | | |

| **Supplementary Table 9 SNPs from the Covid-Host Genetics Initiative to predict COVID-19 severity (B2) for reverse MR analyses** | | | | | | | | | | | | | |
| --- | --- | --- | --- | --- | --- | --- | --- | --- | --- | --- | --- | --- | --- |
| SNP  (gene) | Chr | BP(hg19) | EA | OA | EAF | Beta | SE | P-value | R^2^ | F statistic | Proxy SNP | r^2^ | Study |
| rs10490770  (LZTFL1) | 3 | 45864732 | C | T | 0.08 | 0.499 | 0.029 | 2.81E-64 | 0.03665 | 71814.597 | NA | 1.00 | COVID-19 HGI. 2021 |
| rs529565  (ABO) | 9 | 136149500 | T | C | 0.65 | -0.126 | 0.020 | 2.50E-10 | 0.00722 | 13650.402 | NA | 1.00 | COVID-19 HGI. 2021 |
| rs10774671  (OAS1) | 12 | 113357193 | A | G | 0.67 | 0.116 | 0.019 | 8.83E-10 | 0.00595 | 11298.781 | NA | 1.00 | COVID-19 HGI. 2021 |
| rs1819040 (KANSL1) | 17 | 44221350 | A | T | 0.19 | -0.120 | 0.021 | 2.07E-08 | 0.00443 | 7178.476 | rs2532307 | 1.00 | COVID-19 HGI. 2021 |
| rs2109069  (DPP9) | 19 | 4719443 | A | G | 0.32 | 0.144 | 0.019 | 2.72E-14 | 0.00902 | 17157.824 | NA | 1.00 | COVID-19 HGI. 2021 |
| rs13050728 (IFNAR2) | 21 | 34617729 | C | T | 0.66 | -0.166 | 0.019 | 3.73E-18 | 0.01237 | 23602.223 | rs2252639 | 1.00 | COVID-19 HGI. 2021 |
| Chr: Chromosome; EA: Effect allele; OA: Other allele; EAF: Effect allele frequency; SE: Standard error | | | | | | | | | | | | | |

| **Supplementary Table 10 SNPs from the Covid-Host Genetics Initiative to predict COVID-19 severity (A2) for reverse MR analyses** | | | | | | | | | | | | | |
| --- | --- | --- | --- | --- | --- | --- | --- | --- | --- | --- | --- | --- | --- |
| SNP(gene) | Chr | BP(hg19) | EA | OA | EAF | Beta | SE | P-value | R^2^ | F statistic | Proxy SNP | r^2^ | Study |
| rs10490770  (LZTFL1) | 3 | 45864732 | C | T | 0.08 | 0.625 | 0.040 | 7.86E-55 | 0.05750 | 84699.788 | NA | 1.00 | COVID-19 HGI. 2021 |
| rs10774671  (OAS1) | 12 | 113357193 | A | G | 0.65 | 0.191 | 0.027 | 2.40E-12 | 0.01660 | 23435.473 | NA | 1.00 | COVID-19 HGI. 2021 |
| rs77534576  (TAC4) | 17 | 47940666 | A | C | 0.03 | 0.408 | 0.070 | 4.27E-09 | 0.00969 | 13584.650 | rs3848456 | 0.85 | COVID-19 HGI. 2021 |
| rs2109069  (DPP9) | 19 | 4719443 | A | G | 0.32 | 0.237 | 0.026 | 5.86E-20 | 0.02444 | 34781.079 | NA | 1.00 | COVID-19 HGI. 2021 |
| rs13050728  (IFNAR2) | 21 | 34615210 | G | A | 0.67 | -0.192 | 0.026 | 2.71E-13 | 0.01630 | 23004.922 | rs2300370 | 1.00 | COVID-19 HGI. 2021 |
| Chr: Chromosome; EA: Effect allele; OA: Other allele; EAF: Effect allele frequency; SE: Standard error | | | | | | | | | | | | | |

| **Supplementary Table 11 Variance in LTL explained by different SNP sets and F-statistics for MR primary and sensitivity analysis** | | | | | | | | | | | | | | | |
| --- | --- | --- | --- | --- | --- | --- | --- | --- | --- | --- | --- | --- | --- | --- | --- |
| Group | 20 SNP set | | | 18 SNP set | | | 12 SNP set | | | 52 SNP set | | | 16 SNP set | | |
|  | N | R^2^ | F statistic | N | R^2^ | F statistic | N | R^2^ | F statistic | N | R^2^ | F statistic | N | R^2^ | F statistic |
| COVID-19  Susceptibility(C2) | 20 | 0.01773 | 70.913 | 18 | 0.01665 | 73.909 | 12 | 0.01368 | 90.831 | 45 | 0.02647 | 47.459 | 16 | 0.03566 | 21.200 |
| COVID-19  Susceptibility(C1) | 20 | 0.01773 | 70.913 | 18 | 0.01665 | 73.909 | 12 | 0.01368 | 90.831 | 45 | 0.02647 | 47.459 | 16 | 0.03566 | 21.200 |
| COVID-19  Susceptibility(D1) | 20 | 0.01773 | 70.913 | 18 | 0.01665 | 73.909 | 12 | 0.01368 | 90.831 | 46 | 0.02697 | 47.328 | 16 | 0.03566 | 21.200 |
| COVID-19  Severity(B2) | 20 | 0.01773 | 70.913 | 18 | 0.01665 | 73.909 | 12 | 0.01368 | 90.831 | 44 | 0.02614 | 47.917 | 16 | 0.03566 | 21.200 |
| COVID-19  Severity(B1) | 20 | 0.01773 | 70.913 | 18 | 0.01665 | 73.909 | 12 | 0.01368 | 90.831 | 44 | 0.02614 | 47.917 | 16 | 0.03566 | 21.200 |
| COVID-19  Severity(A1) | 20 | 0.01773 | 70.913 | 18 | 0.01665 | 73.909 | 12 | 0.01368 | 90.831 | 45 | 0.02647 | 47.459 | 16 | 0.03566 | 21.200 |
| COVID-19  Severity(A2) | 20 | 0.01773 | 70.913 | 18 | 0.01665 | 73.909 | 12 | 0.01368 | 90.831 | 45 | 0.02647 | 47.459 | 16 | 0.03566 | 21.200 |

**Supplementary Table 12 Results of** **forward MR analyses and sensitivity analyses**

| Full analysis with all SNPs (20 SNP set) | | | | | | | | | | | |
| --- | --- | --- | --- | --- | --- | --- | --- | --- | --- | --- | --- |
| **Exposure** | **Outcome** | **Method** | **Beta** | **OR** | **95% CI** | **SE** | **P value^*^** | **Heterogeneity P** | **Intercept**  **P** | **I^2^(%)** | **MR-Presso**  **P** |
| LTL | COVID-19 Susceptibility(C2) | Inverse variance weighted | -0.065 | 0.94 | 0.85 to 1.04 | 0.051 | 0.202 | 0.259 | NA | NA | 0.313 |
|  |  | MR Egger | -0.213 | 0.81 | 0.59 to 1.10 | 0.159 | 0.199 | 0.262 | 0.337 | 98.56 |  |
|  |  | MR Egger (bootstrap) | -0.108 | 0.90 | 0.73 to 1.10 | 0.109 | 0.146 | NA | NA | NA |  |
|  |  | Penalised weighted median | -0.079 | 0.92 | 0.80 to 1.07 | 0.075 | 0.293 | NA | NA | NA |  |
|  |  | Simple mode | -0.179 | 0.84 | 0.65 to 1.07 | 0.125 | 0.167 | NA | NA | NA |  |
|  |  | Weighted median | -0.078 | 0.93 | 0.80 to 1.07 | 0.073 | 0.286 | NA | NA | NA |  |
|  |  | Weighted mode | -0.098 | 0.91 | 0.77 to 1.07 | 0.085 | 0.266 | NA | NA | NA |  |
| LTL | Severity(B2) | Inverse variance weighted | -0.161 | 0.85 | 0.70 to 1.03 | 0.097 | 0.099 | 0.149 | NA | NA | 0.182 |
|  |  | MR Egger | -0.165 | 0.85 | 0.44 to 1.62 | 0.330 | 0.623 | 0.115 | 0.989 | 98.54 |  |
|  |  | MR Egger (bootstrap) | -0.129 | 0.88 | 0.59 to 1.34 | 0.217 | 0.286 | NA | NA | NA |  |
|  |  | Penalised weighted median | -0.159 | 0.85 | 0.64 to 1.13 | 0.143 | 0.267 | NA | NA | NA |  |
|  |  | Simple mode | -0.113 | 0.89 | 0.56 to 1.41 | 0.234 | 0.634 | NA | NA | NA |  |
|  |  | Weighted median | -0.158 | 0.85 | 0.65 to 1.12 | 0.139 | 0.255 | NA | NA | NA |  |
|  |  | Weighted mode | -0.113 | 0.89 | 0.65 to 1.23 | 0.165 | 0.501 | NA | NA | NA |  |
| Sensitivity analysis excluding SNPs associated with hypertension and cerebral infarction identified by PhenoScanner (18 SNP set) | | | | | | | | | | | |
| **Exposure** | **Outcome** | **Method** | **Beta** | **OR** | **95% CI** | **SE** | **P value^*^** | **Heterogeneity P** | **Intercept**  **P** | **I^2^(%)** | **MR-Presso**  **P** |
| LTL | Susceptibility(C2) | Inverse variance weighted | -0.045 | 0.96 | 0.86 to 1.06 | 0.053 | 0.402 | 0.259 | NA | NA | 0.299 |
|  |  | MR Egger | -0.198 | 0.82 | 0.58 to 1.16 | 0.178 | 0.283 | 0.253 | 0.375 | 98.62 |  |
|  |  | MR Egger (bootstrap) | -0.126 | 0.88 | 0.70 to 1.11 | 0.116 | 0.118 | NA | NA | NA |  |
|  |  | Penalised weighted median | -0.070 | 0.93 | 0.80 to 1.08 | 0.075 | 0.354 | NA | NA | NA |  |
|  |  | Simple mode | 0.038 | 1.04 | 0.81 to 1.34 | 0.129 | 0.772 | NA | NA | NA |  |
|  |  | Weighted median | -0.069 | 0.93 | 0.81 to 1.08 | 0.075 | 0.359 | NA | NA | NA |  |
|  |  | Weighted mode | -0.080 | 0.92 | 0.79 to 1.08 | 0.082 | 0.340 | NA | NA | NA |  |
| LTL | Severity(B2) | Inverse variance weighted | -0.150 | 0.86 | 0.71 to 1.05 | 0.101 | 0.137 | 0.101 | NA | NA | 0.117 |
|  |  | MR Egger | -0.235 | 0.79 | 0.38 to 1.66 | 0.378 | 0.543 | 0.077 | 0.814 | 98.61 |  |
|  |  | MR Egger (bootstrap) | -0.133 | 0.88 | 0.57 to 1.34 | 0.264 | 0.264 | NA | NA | NA |  |
|  |  | Penalised weighted median | -0.140 | 0.87 | 0.65 to 1.16 | 0.148 | 0.343 | NA | NA | NA |  |
|  |  | Simple mode | -0.063 | 0.94 | 0.59 to 1.49 | 0.236 | 0.792 | NA | NA | NA |  |
|  |  | Weighted median | -0.141 | 0.87 | 0.66 to 1.15 | 0.144 | 0.327 | NA | NA | NA |  |
|  |  | Weighted mode | -0.104 | 0.90 | 0.63 to 1.28 | 0.179 | 0.567 | NA | NA | NA |  |
| Sensitivity analysis restricted to gene in Telomere Biology (12 SNP set) | | | | | | | | | | | |
| **Exposure** | **Outcome** | **Method** | **Beta** | **OR** | **95% CI** | **SE** | **P value^*^** | **Heterogeneity P** | **Intercept**  **P** | **I^2^(%)** | **MR-Presso**  **P** |
| LTL | Susceptibility(C2) | Inverse variance weighted | -0.077 | 0.93 | 0.83 to 1.04 | 0.058 | 0.183 | 0.154 | NA | NA | 0.213 |
|  |  | MR Egger | -0.279 | 0.76 | 0.51 to 1.12 | 0.198 | 0.190 | 0.173 | 0.303 | 98.91 |  |
|  |  | MR Egger (bootstrap) | -0.134 | 0.87 | 0.68 to 1.12 | 0.127 | 0.148 | NA | NA | NA |  |
|  |  | Penalised weighted median | -0.085 | 0.92 | 0.79 to 1.07 | 0.077 | 0.266 | NA | NA | NA |  |
|  |  | Simple mode | -0.118 | 0.89 | 0.70 to 1.13 | 0.121 | 0.350 | NA | NA | NA |  |
|  |  | Weighted median | -0.084 | 0.92 | 0.79 to 1.07 | 0.076 | 0.271 | NA | NA | NA |  |
|  |  | Weighted mode | -0.093 | 0.91 | 0.76 to 1.09 | 0.090 | 0.322 | NA | NA | NA |  |
| LTL | Severity(B2) | Inverse variance weighted | -0.120 | 0.89 | 0.71 to 1.10 | 0.112 | 0.284 | 0.057 | NA | NA | 0.068 |
|  |  | MR Egger | -0.321 | 0.73 | 0.30 to 1.73 | 0.443 | 0.485 | 0.043 | 0.639 | 98.91 |  |
|  |  | MR Egger (bootstrap) | -0.192 | 0.83 | 0.51 to 1.34 | 0.247 | 0.226 | NA | NA | NA |  |
|  |  | Penalised weighted median | -0.185 | 0.83 | 0.61 to 1.13 | 0.158 | 0.242 | NA | NA | NA |  |
|  |  | Simple mode | -0.247 | 0.78 | 0.48 to 1.26 | 0.245 | 0.335 | NA | NA | NA |  |
|  |  | Weighted median | -0.182 | 0.83 | 0.62 to 1.13 | 0.155 | 0.242 | NA | NA | NA |  |
|  |  | Weighted mode | -0.170 | 0.84 | 0.60 to 1.19 | 0.177 | 0.358 | NA | NA | NA |  |
| Sensitivity analysis including more SNPs associated with LTL at a liberal significance level (FDR<0.05) (52 SNP set) | | | | | | | | | | | |
| **Exposure** | **Outcome** | **Method** | **Beta** | **OR** | **95% CI** | **SE** | **P value^*^** | **Heterogeneity P** | **Intercept**  **P** | **I^2^(%)** | **MR-Presso**  **P** |
| LTL | Susceptibility(C2) | Inverse variance weighted | -0.066 | 0.94 | 0.87 to 1.01 | 0.038 | 0.087 | 0.525 | NA | NA | 0.538 |
|  |  | MR Egger | -0.057 | 0.94 | 0.79 to 1.13 | 0.091 | 0.535 | 0.482 | 0.915 | 0.9768 |  |
|  |  | MR Egger (bootstrap) | -0.081 | 0.92 | 0.81 to 1.05 | 0.068 | 0.112 | NA | NA | NA |  |
|  |  | Penalised weighted median | -0.064 | 0.94 | 0.84 to 1.05 | 0.058 | 0.271 | NA | NA | NA |  |
|  |  | Simple mode | -0.172 | 0.84 | 0.68 to 1.04 | 0.106 | 0.111 | NA | NA | NA |  |
|  |  | Weighted median | -0.061 | 0.94 | 0.84 to 1.05 | 0.057 | 0.280 | NA | NA | NA |  |
|  |  | Weighted mode | -0.087 | 0.92 | 0.81 to 1.04 | 0.063 | 0.174 | NA | NA | NA |  |
| LTL | Severity(B2) | Inverse variance weighted | -0.129 | 0.88 | 0.76 to 1.02 | 0.076 | 0.090 | 0.143 | NA | NA | 0.164 |
|  |  | MR Egger | 0.140 | 1.15 | 0.78 to 1.70 | 0.199 | 0.486 | 0.179 | 0.145 | 0.9772 |  |
|  |  | MR Egger (bootstrap) | -0.042 | 0.96 | 0.74 to 1.24 | 0.130 | 0.364 | NA | NA | NA |  |
|  |  | Penalised weighted median | -0.123 | 0.88 | 0.70 to 1.11 | 0.117 | 0.294 | NA | NA | NA |  |
|  |  | Simple mode | -0.013 | 0.99 | 0.65 to 1.50 | 0.215 | 0.951 | NA | NA | NA |  |
|  |  | Weighted median | -0.123 | 0.88 | 0.70 to 1.12 | 0.119 | 0.299 | NA | NA | NA |  |
|  |  | Weighted mode | -0.043 | 0.96 | 0.76 to 1.20 | 0.116 | 0.715 | NA | NA | NA |  |
| Sensitivity analysis including SNPs utilized by Haycock et al (16 SNP set) | | | | | | | | | | | |
| **Exposure** | **Outcome** | **Method** | **Beta** | **OR** | **95% CI** | **SE** | **P value^*^** | **Heterogeneity P** | **Intercept**  **P** | **I^2^(%)** | **MR-Presso**  **P** |
| LTL | Susceptibility(C2) | Inverse variance weighted | -0.005 | 0.99 | 0.93 to 1.06 | 0.033 | 0.874 | 0.688 | NA | NA | 0.691 |
|  |  | MR Egger | 0.054 | 1.06 | 0.85 to 1.31 | 0.110 | 0.633 | 0.641 | 0.583 | 92.37 |  |
|  |  | MR Egger (bootstrap) | -0.096 | 0.91 | 0.80 to 1.03 | 0.064 | 0.065 | NA | NA | NA |  |
|  |  | Penalised weighted median | -0.025 | 0.98 | 0.89 to 1.06 | 0.044 | 0.571 | NA | NA | NA |  |
|  |  | Simple mode | -0.001 | 1.00 | 0.89 to 1.12 | 0.057 | 0.983 | NA | NA | NA |  |
|  |  | Weighted median | -0.025 | 0.98 | 0.90 to 1.06 | 0.042 | 0.552 | NA | NA | NA |  |
|  |  | Weighted mode | 0.002 | 1.00 | 0.91 to 1.11 | 0.051 | 0.968 | NA | NA | NA |  |
| LTL | Severity(B2) | Inverse variance weighted | 0.026 | 1.03 | 0.91 to 1.16 | 0.064 | 0.685 | 0.273 | NA | NA | 0.322 |
|  |  | MR Egger | 0.283 | 1.33 | 0.84 to 2.09 | 0.231 | 0.241 | 0.300 | 0.264 | 93.23 |  |
|  |  | MR Egger (bootstrap) | -0.034 | 0.97 | 0.76 to 1.23 | 0.122 | 0.380 | NA | NA | NA |  |
|  |  | Penalised weighted median | 0.029 | 1.03 | 0.87 to 1.21 | 0.085 | 0.736 | NA | NA | NA |  |
|  |  | Simple mode | -0.031 | 0.97 | 0.76 to 1.23 | 0.123 | 0.804 | NA | NA | NA |  |
|  |  | Weighted median | 0.025 | 1.02 | 0.87 to 1.21 | 0.086 | 0.774 | NA | NA | NA |  |
|  |  | Weighted mode | -0.011 | 0.99 | 0.80 to 1.22 | 0.106 | 0.922 | NA | NA | NA |  |

*P values shown have not been corrected/adjusted for multiple comparisons

**Supplementary Table 13 Results of forward MR analyses for different definition of COVID-19 Susceptibility or Severity**

| Full analysis with all SNPs (20 SNP set) | | | | | | | | | | | |  |  |
| --- | --- | --- | --- | --- | --- | --- | --- | --- | --- | --- | --- | --- | --- |
| **Exposure** | **Outcome** | **Method** | **Beta** | **OR** | **95% CI** | **SE** | **P value^*^** | **Heterogeneity P** | **Intercept**  **P** | **I^2^(%)** | **MR-Presso**  **P** |  |  |
| LTL | Susceptibility(C1) | Inverse variance weighted | -0.111 | 0.89 | 0.76 to 1.06 | 0.085 | 0.190 | 0.392 | NA | NA | 0.444 |  |  |
|  |  | MR Egger | -0.247 | 0.78 | 0.47 to 1.29 | 0.255 | 0.346 | 0.351 | 0.576 | 98.55 |  |  |  |
|  |  | MR Egger (bootstrap) | -0.051 | 0.95 | 0.66 to 1.37 | 0.187 | 0.397 | NA | NA | NA |  |  |  |
|  |  | Penalised weighted median | -0.100 | 0.90 | 0.72 to 1.14 | 0.116 | 0.389 | NA | NA | NA |  |  |  |
|  |  | Simple mode | -0.007 | 0.99 | 0.70 to 1.41 | 0.180 | 0.969 | NA | NA | NA |  |  |  |
|  |  | Weighted median | -0.110 | 0.90 | 0.71 to 1.13 | 0.117 | 0.349 | NA | NA | NA |  |  |  |
|  |  | Weighted mode | -0.091 | 0.91 | 0.69 to 1.21 | 0.142 | 0.530 | NA | NA | NA |  |  |  |
| LTL | Susceptibility(D1) | Inverse variance weighted | 0.048 | 1.05 | 0.80 to 1.38 | 0.141 | 0.733 | 0.178 | NA | NA | 0.200 |  |  |
|  |  | MR Egger | -0.389 | 0.68 | 0.27 to 1.69 | 0.465 | 0.414 | 0.183 | 0.330 | 98.56 |  |  |  |
|  |  | MR Egger (bootstrap) | -0.177 | 0.84 | 0.48 to 1.47 | 0.288 | 0.291 | NA | NA | NA |  |  |  |
|  |  | Penalised weighted median | -0.162 | 0.85 | 0.57 to 1.26 | 0.201 | 0.419 | NA | NA | NA |  |  |  |
|  |  | Simple mode | -0.292 | 0.75 | 0.41 to 1.37 | 0.308 | 0.355 | NA | NA | NA |  |  |  |
|  |  | Weighted median | -0.138 | 0.87 | 0.59 to 1.30 | 0.203 | 0.497 | NA | NA | NA |  |  |  |
|  |  | Weighted mode | -0.173 | 0.84 | 0.55 to 1.29 | 0.220 | 0.440 | NA | NA | NA |  |  |  |
| LTL | Severity(B1) | Inverse variance weighted | -0.131 | 0.88 | 0.63 to 1.21 | 0.165 | 0.426 | 0.849 | NA | NA | 0.854 |  |  |
|  |  | MR Egger | -0.111 | 0.89 | 0.47 to 1.71 | 0.331 | 0.359 | 0.818 | 0.634 | 98.56 |  |  |  |
|  |  | MR Egger (bootstrap) | 0.081 | 1.08 | 0.43 to 2.72 | 0.469 | 0.864 | NA | NA | NA |  |  |  |
|  |  | Penalised weighted median | -0.139 | 0.87 | 0.54 to 1.39 | 0.239 | 0.560 | NA | NA | NA |  |  |  |
|  |  | Simple mode | -0.337 | 0.71 | 0.33 to 1.53 | 0.389 | 0.397 | NA | NA | NA |  |  |  |
|  |  | Weighted median | -0.139 | 0.87 | 0.55 to 1.37 | 0.232 | 0.548 | NA | NA | NA |  |  |  |
|  |  | Weighted mode | -0.148 | 0.86 | 0.47 to 1.58 | 0.308 | 0.636 | NA | NA | NA |  |  |  |
| LTL | Severity(A1) | Inverse variance weighted | -1.035 | 0.36 | 0.08 to 1.68 | 0.793 | 0.192 | 0.547 | NA | NA | 0.550 |  |  |
|  |  | MR Egger | 2.917 | 18.48 | 0.20 to 1631.50 | 2.286 | 0.218 | 0.713 | 0.082 | 98.55 |  |  |  |
|  |  | MR Egger (bootstrap) | 0.682 | 1.98 | 0.11 to 35.41 | 1.472 | 0.321 | NA | NA | NA |  |  |  |
|  |  | Penalised weighted median | -0.783 | 0.46 | 0.05 to 4.35 | 1.150 | 0.496 | NA | NA | NA |  |  |  |
|  |  | Simple mode | -0.597 | 0.55 | 0.02 to 20.15 | 1.837 | 0.749 | NA | NA | NA |  |  |  |
|  |  | Weighted median | -0.783 | 0.46 | 0.05 to 4.00 | 1.107 | 0.479 | NA | NA | NA |  |  |  |
|  |  | Weighted mode | -0.229 | 0.79 | 0.05 to 13.00 | 1.426 | 0.874 | NA | NA | NA |  |  |  |
| LTL | Severity(A2) | Inverse variance weighted | -0.075 | 0.93 | 0.69 to 1.24 | 0.149 | 0.616 | 0.347 | NA | NA | 0.360 |  |  |
|  |  | MR Egger | -0.013 | 0.99 | 0.41 to 2.38 | 0.449 | 0.978 | 0.290 | 0.884 | 98.54 |  |  |  |
|  |  | MR Egger (bootstrap) | -0.053 | 0.95 | 0.56 to 1.61 | 0.269 | 0.421 | NA | NA | NA |  |  |  |
|  |  | Penalised weighted median | -0.239 | 0.79 | 0.52 to 1.19 | 0.212 | 0.261 | NA | NA | NA |  |  |  |
|  |  | Simple mode | -0.338 | 0.71 | 0.33 to 1.56 | 0.401 | 0.409 | NA | NA | NA |  |  |  |
|  |  | Weighted median | -0.239 | 0.79 | 0.51 to 1.21 | 0.217 | 0.272 | NA | NA | NA |  |  |  |
|  |  | Weighted mode | -0.231 | 0.79 | 0.48 to 1.32 | 0.259 | 0.384 | NA | NA | NA |  |  |  |
| Sensitivity analysis including more SNPs associated with LTL at a liberal significance level(FDR<0.05) (52 SNP set) | | | | | | | | | | | |  |  |
| **Exposure** | **Outcome** | **Method** | **Beta** | **OR** | **95% CI** | **SE** | **P value^*^** | **Heterogeneity P** | **Intercept**  **P** | **I^2^(%)** | **MR-Presso**  **P** |  |  |
| LTL | Susceptibility(C1) | Inverse variance weighted | -0.124 | 0.88 | 0.77 to 1.01 | 0.070 | 0.073 | 0.201 | NA | NA | 0.239 |  |  |
|  |  | MR Egger | -0.069 | 0.93 | 0.65 to 1.35 | 0.187 | 0.713 | 0.176 | 0.748 | 97.69 |  |  |  |
|  |  | MR Egger (bootstrap) | 0.019 | 1.02 | 0.76 to 1.36 | 0.148 | 0.435 | NA | NA | NA |  |  |  |
|  |  | Penalised weighted median | -0.076 | 0.93 | 0.76 to 1.13 | 0.100 | 0.446 | NA | NA | NA |  |  |  |
|  |  | Simple mode | -0.084 | 0.92 | 0.62 to 1.36 | 0.199 | 0.674 | NA | NA | NA |  |  |  |
|  |  | Weighted median | -0.096 | 0.91 | 0.74 to 1.12 | 0.108 | 0.374 | NA | NA | NA |  |  |  |
|  |  | Weighted mode | -0.076 | 0.93 | 0.71 to 1.20 | 0.133 | 0.569 | NA | NA | NA |  |  |  |
| LTL | Susceptibility(D1) | Inverse variance weighted | 0.014 | 1.01 | 0.80 to 1.28 | 0.118 | 0.909 | 0.082 | NA | NA | 0.073 |  |  |
|  |  | MR Egger | -0.106 | 0.90 | 0.47 to 1.73 | 0.335 | 0.752 | 0.070 | 0.697 | 97.66 |  |  |  |
|  |  | MR Egger (bootstrap) | -0.025 | 0.97 | 0.63 to 1.51 | 0.223 | 0.460 | NA | NA | NA |  |  |  |
|  |  | Penalised weighted median | -0.057 | 0.94 | 0.66 to 1.35 | 0.181 | 0.751 | NA | NA | NA |  |  |  |
|  |  | Simple mode | 0.482 | 1.62 | 0.84 to 3.14 | 0.337 | 0.160 | NA | NA | NA |  |  |  |
|  |  | Weighted median | -0.051 | 0.95 | 0.67 to 1.36 | 0.182 | 0.781 | NA | NA | NA |  |  |  |
|  |  | Weighted mode | -0.089 | 0.91 | 0.61 to 1.38 | 0.208 | 0.672 | NA | NA | NA |  |  |  |
| LTL | Severity(B1) | Inverse variance weighted | 0.073 | 1.08 | 0.82 to 1.41 | 0.137 | 0.597 | 0.945 | NA | NA | 0.947 |  |  |
|  |  | MR Egger | 0.037 | 1.04 | 0.53 to 2.03 | 0.343 | 0.914 | 0.931 | 0.911 | 97.72 |  |  |  |
|  |  | MR Egger (bootstrap) | 0.082 | 1.09 | 0.64 to 1.85 | 0.271 | 0.372 | NA | NA | NA |  |  |  |
|  |  | Penalised weighted median | 0.014 | 1.01 | 0.68 to 1.52 | 0.207 | 0.945 | NA | NA | NA |  |  |  |
|  |  | Simple mode | 0.035 | 1.04 | 0.50 to 2.15 | 0.373 | 0.926 | NA | NA | NA |  |  |  |
|  |  | Weighted median | 0.014 | 1.01 | 0.68 to 1.51 | 0.203 | 0.944 | NA | NA | NA |  |  |  |
|  |  | Weighted mode | 0.090 | 1.09 | 0.64 to 1.87 | 0.274 | 0.744 | NA | NA | NA |  |  |  |
| LTL | Severity(A1) | Inverse variance weighted | 0.873 | 2.39 | 0.66 to 8.72 | 0.660 | 0.186 | 0.603 | NA | NA | 0.656 |  |  |
|  |  | MR Egger | -0.686 | 0.50 | 0.02 to 12.32 | 1.631 | 0.676 | 0.608 | 0.302 | 97.69 |  |  |  |
|  |  | MR Egger (bootstrap) | 0.057 | 1.06 | 0.11 to 10.16 | 1.154 | 0.473 | NA | NA | NA |  |  |  |
|  |  | Penalised weighted median | 0.285 | 1.33 | 0.19 to 9.19 | 0.986 | 0.773 | NA | NA | NA |  |  |  |
|  |  | Simple mode | 0.401 | 1.49 | 0.03 to 69.88 | 1.962 | 0.839 | NA | NA | NA |  |  |  |
|  |  | Weighted median | 0.305 | 1.36 | 0.19 to 9.73 | 1.005 | 0.762 | NA | NA | NA |  |  |  |
|  |  | Weighted mode | 0.091 | 1.10 | 0.09 to 12.72 | 1.251 | 0.942 | NA | NA | NA |  |  |  |
| LTL | Severity(A2) | Inverse variance weighted | -0.094 | 0.91 | 0.71 to 1.16 | 0.124 | 0.449 | 0.535 | NA | NA | 0.529 |  |  |
|  |  | MR Egger | -0.036 | 0.96 | 0.62 to 1.50 | 0.225 | 0.433 | 0.495 | 0.803 | 97.69 |  |  |  |
|  |  | MR Egger (bootstrap) | -0.163 | 0.85 | 0.47 to 1.54 | 0.303 | 0.593 | NA | NA | NA |  |  |  |
|  |  | Penalised weighted median | -0.279 | 0.76 | 0.52 to 1.09 | 0.188 | 0.137 | NA | NA | NA |  |  |  |
|  |  | Simple mode | 0.264 | 1.30 | 0.60 to 2.81 | 0.392 | 0.504 | NA | NA | NA |  |  |  |
|  |  | Weighted median | -0.249 | 0.78 | 0.54 to 1.13 | 0.190 | 0.190 | NA | NA | NA |  |  |  |
|  |  | Weighted mode | -0.026 | 0.97 | 0.59 to 1.62 | 0.260 | 0.920 | NA | NA | NA |  |  |  |
| Sensitivity analysis including SNPs utilized by Haycock et al (16 SNP set) | | | | | | | | | | | |  | Weighted mode |
| **Exposure** | **Outcome** | **Method** | **Beta** | **OR** | **95% CI** | **SE** | **P value^*^** | **Heterogeneity P** | **Intercept**  **P** | **I^2^(%)** | **MR-Presso**  **P** |  |  |
| LTL | Susceptibility(C1) | Inverse variance weighted | 0.005 | 1.00 | 0.90 to 1.12 | 0.054 | 0.928 | 0.952 | NA | NA | 0.944 |  |  |
|  |  | MR Egger | -0.009 | 0.99 | 0.70 to 1.40 | 0.176 | 0.962 | 0.928 | 0.937 | 92.76 |  |  |  |
|  |  | MR Egger (bootstrap) | 0.030 | 1.03 | 0.83 to 1.27 | 0.107 | 0.406 | NA | NA | NA |  |  |  |
|  |  | Penalised weighted median | 0.036 | 1.04 | 0.90 to 1.19 | 0.070 | 0.612 | NA | NA | NA |  |  |  |
|  |  | Simple mode | 0.030 | 1.03 | 0.86 to 1.24 | 0.093 | 0.752 | NA | NA | NA |  |  |  |
|  |  | Weighted median | 0.036 | 1.04 | 0.90 to 1.19 | 0.072 | 0.622 | NA | NA | NA |  |  |  |
|  |  | Weighted mode | 0.034 | 1.03 | 0.87 to 1.23 | 0.089 | 0.710 | NA | NA | NA |  |  |  |
| LTL | Susceptibility(D1) | Inverse variance weighted | -0.181 | 0.83 | 0.69 to 1.01 | 0.095 | 0.056 | 0.998 | NA | NA | 0.999 |  |  |
|  |  | MR Egger | 0.078 | 1.08 | 0.60 to 1.94 | 0.299 | 0.798 | 0.999 | 0.377 | 91.75 |  |  |  |
|  |  | MR Egger (bootstrap) | -0.164 | 0.85 | 0.59 to 1.22 | 0.187 | 0.193 | NA | NA | NA |  |  |  |
|  |  | Penalised weighted median | -0.198 | 0.82 | 0.65 to 1.04 | 0.121 | 0.102 | NA | NA | NA |  |  |  |
|  |  | Simple mode | -0.204 | 0.82 | 0.59 to 1.14 | 0.169 | 0.247 | NA | NA | NA |  |  |  |
|  |  | Weighted median | -0.198 | 0.82 | 0.65 to 1.04 | 0.121 | 0.102 | NA | NA | NA |  |  |  |
|  |  | Weighted mode | -0.191 | 0.83 | 0.61 to 1.11 | 0.152 | 0.229 | NA | NA | NA |  |  |  |
| LTL | Severity(B1) | Inverse variance weighted | -0.027 | 0.97 | 0.79 to 1.20 | 0.108 | 0.800 | 0.511 | NA | NA | 0.541 |  |  |
|  |  | MR Egger | 0.061 | 1.06 | 0.70 to 1.62 | 0.216 | 0.381 | 0.542 | 0.257 | 92.43 |  |  |  |
|  |  | MR Egger (bootstrap) | 0.377 | 1.46 | 0.72 to 2.95 | 0.359 | 0.312 | NA | NA | NA |  |  |  |
|  |  | Penalised weighted median | 0.107 | 1.11 | 0.84 to 1.48 | 0.146 | 0.464 | NA | NA | NA |  |  |  |
|  |  | Simple mode | 0.111 | 1.12 | 0.75 to 1.66 | 0.202 | 0.590 | NA | NA | NA |  |  |  |
|  |  | Weighted median | 0.097 | 1.10 | 0.83 to 1.47 | 0.147 | 0.511 | NA | NA | NA |  |  |  |
|  |  | Weighted mode | 0.147 | 1.16 | 0.82 to 1.63 | 0.175 | 0.416 | NA | NA | NA |  |  |  |
| LTL | Severity(A1) | Inverse variance weighted | 0.375 | 1.46 | 0.53 to 3.98 | 0.513 | 0.465 | 0.727 | NA | NA | 0.747 |  |  |
|  |  | MR Egger | 1.590 | 4.90 | 0.16 to 152.95 | 1.755 | 0.380 | 0.699 | 0.481 | 93.20 |  |  |  |
|  |  | MR Egger (bootstrap) | 0.560 | 1.75 | 0.24 to 12.51 | 1.003 | 0.277 | NA | NA | NA |  |  |  |
|  |  | Penalised weighted median | 0.257 | 1.29 | 0.33 to 5.03 | 0.693 | 0.711 | NA | NA | NA |  |  |  |
|  |  | Simple mode | 0.146 | 1.16 | 0.14 to 9.89 | 1.095 | 0.896 | NA | NA | NA |  |  |  |
|  |  | Weighted median | 0.257 | 1.29 | 0.33 to 5.02 | 0.692 | 0.710 | NA | NA | NA |  |  |  |
|  |  | Weighted mode | 0.516 | 1.68 | 0.27 to 10.38 | 0.930 | 0.587 | NA | NA | NA |  |  |  |
| LTL | Severity(A2) | Inverse variance weighted | 0.211 | 1.24 | 1.03 to 1.48 | 0.092 | **0.021** | 0.486 | NA | NA | 0.545 |  |  |
|  |  | MR Egger | 0.407 | 1.50 | 0.81 to 2.79 | 0.316 | 0.218 | 0.442 | 0.527 | 94.45 |  |  |  |
|  |  | MR Egger (bootstrap) | 0.056 | 1.06 | 0.75 to 1.49 | 0.173 | 0.370 | NA | NA | NA |  |  |  |
|  |  | Penalised weighted median | 0.279 | 1.32 | 1.05 to 1.67 | 0.120 | **0.020** | NA | NA | NA |  |  |  |
|  |  | Simple mode | 0.229 | 1.26 | 0.94 to 1.67 | 0.146 | 0.137 | NA | NA | NA |  |  |  |
|  |  | Weighted median | 0.275 | 1.32 | 1.04 to 1.67 | 0.121 | **0.023** | NA | NA | NA |  |  |  |
|  |  | Weighted mode | 0.248 | 1.28 | 0.97 to 1.70 | 0.144 | 0.106 | NA | NA | NA |  |  |  |
| *P values shown have not been corrected/adjusted for multiple comparisons | | | | | | | | | | | |  |  |

**
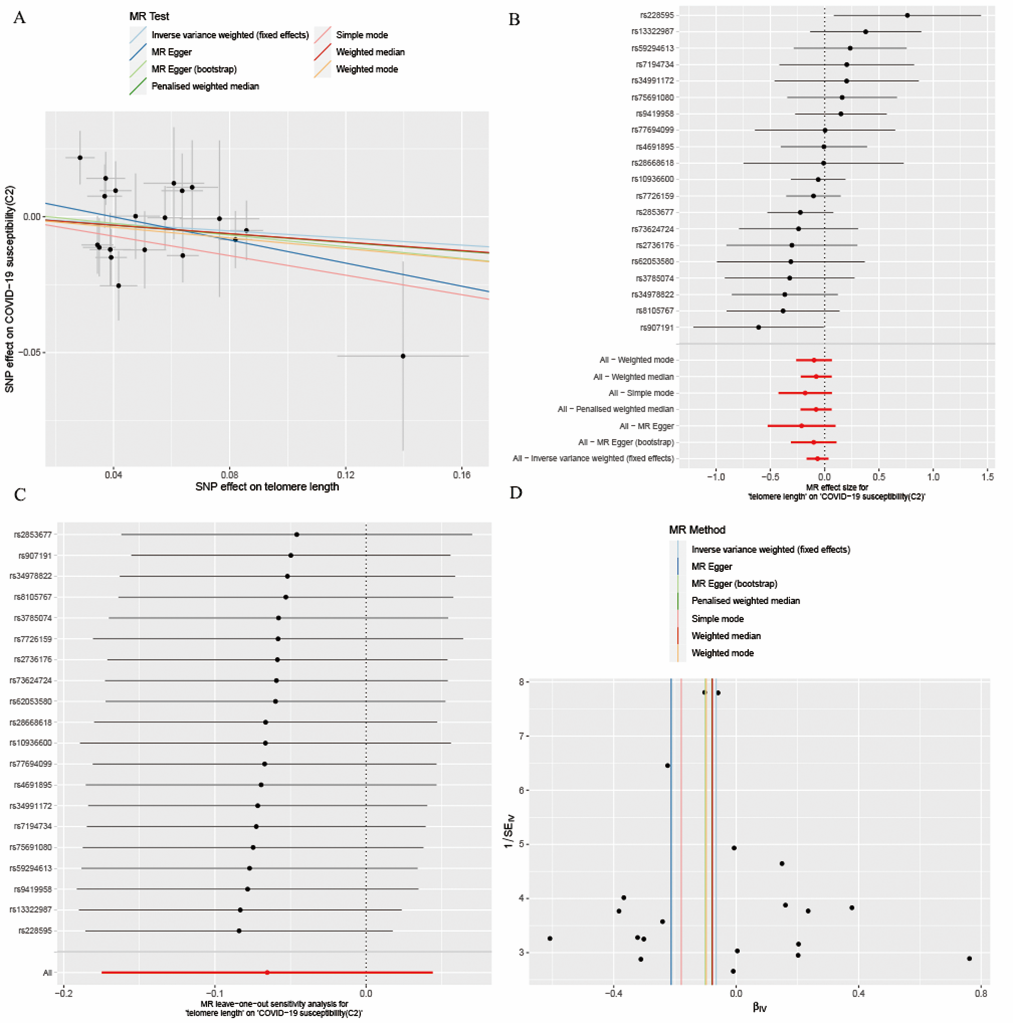
**

**Supplementary Fig. 1 MR Plots for Relationship of Genetically Predicted LTL with COVID-19 Susceptibility (C2).**

**A.** Scatterplot of SNP potential effects on LTL vs COVID-19 Susceptibility (C2), with the slope of each line corresponding to estimated MR effect per method. **B.** Forest plot of individual and combined SNP MR-estimated effect sizes based on seven methods. Data are expressed as raw OR values with 95% CI in 20 SNP set. **C.** Leave-one-out plots for heterogeneous MR LTL-COVID-19 Susceptibility (C2) associations. **D.** Funnel plot of the LTL instrument precision versus the MR effect estimates on the COVID-19 Susceptibility (C2).


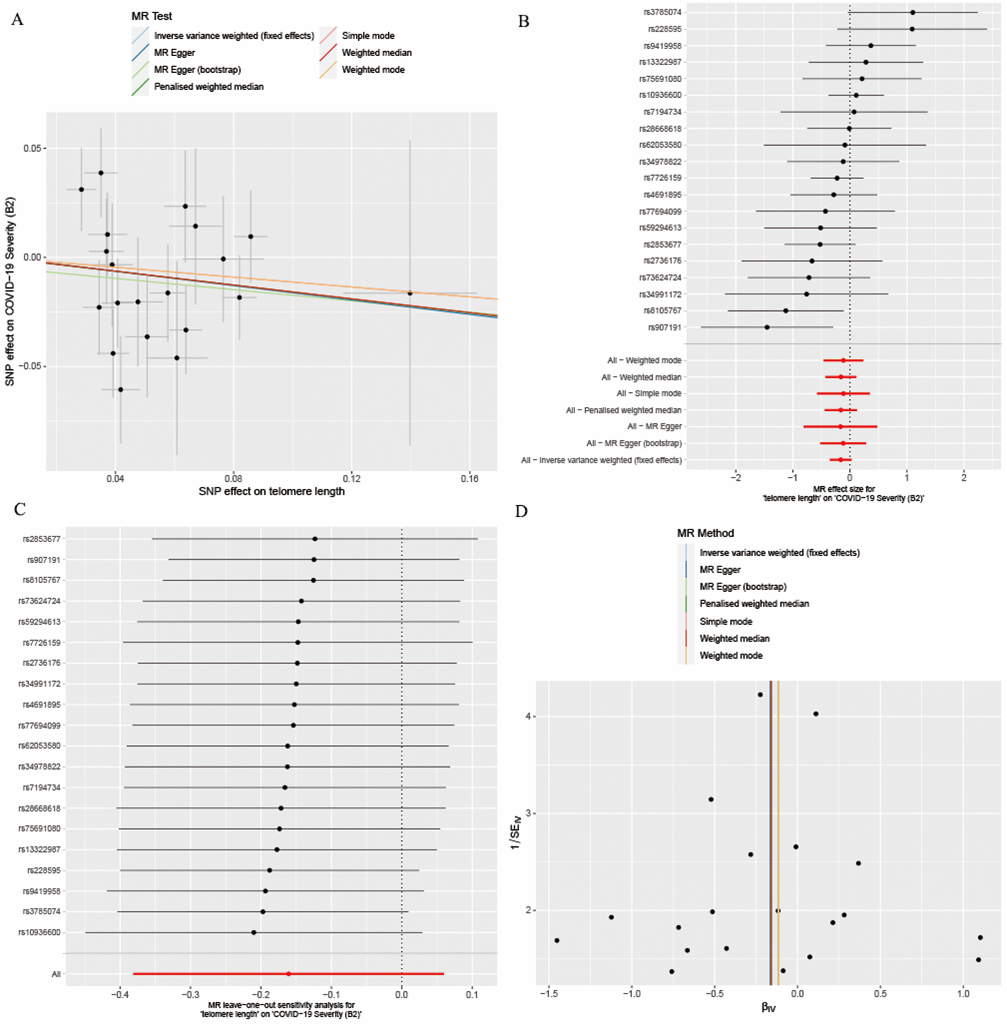


**Supplementary Fig. 2 MR Plots for Relationship of Genetically Predicted LTL with COVID-19 Severity (B2).**

**A.** Scatterplot of SNP potential effects on LTL vs COVID-19 Severity (B2), with the slope of each line corresponding to estimated MR effect per method. **B.** Forest plot of individual and combined SNP MR-estimated effect sizes based on seven methods. Data are expressed as raw OR values with 95% CI in 20 SNP set. **C.** Leave-one-out plots for heterogeneous MR LTL-COVID-19 Severity (B2) associations. **D.** Funnel plot of the LTL instrument precision versus the MR effect estimates on the COVID-19 Severity (B2).


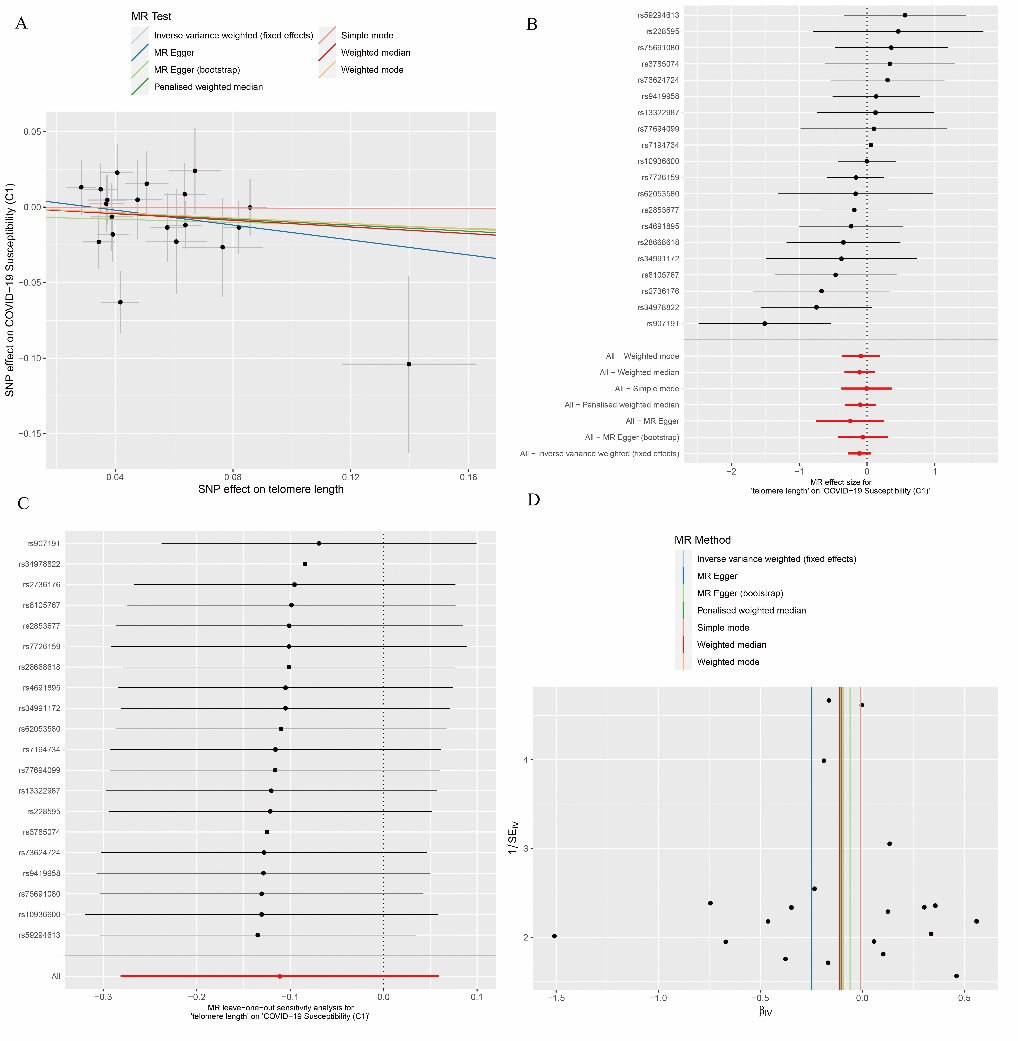


**Supplementary Fig. 3 MR Plots for Relationship of Genetically Predicted LTL with** **COVID-19 Susceptibility (C1).**

**A.** Scatterplot of SNP potential effects on LTL vs COVID-19 Susceptibility (C1), with the slope of each line corresponding to estimated MR effect per method. **B.** Forest plot of individual and combined SNP MR-estimated effect sizes based on seven methods. Data are expressed as raw OR values with 95% CI in 20 SNP set. **C.** Leave-one-out plots for heterogeneous MR LTL-COVID-19 Susceptibility (C1) associations. **D.** Funnel plot of the LTL instrument precision versus the MR effect estimates on the COVID-19 Susceptibility (C1).


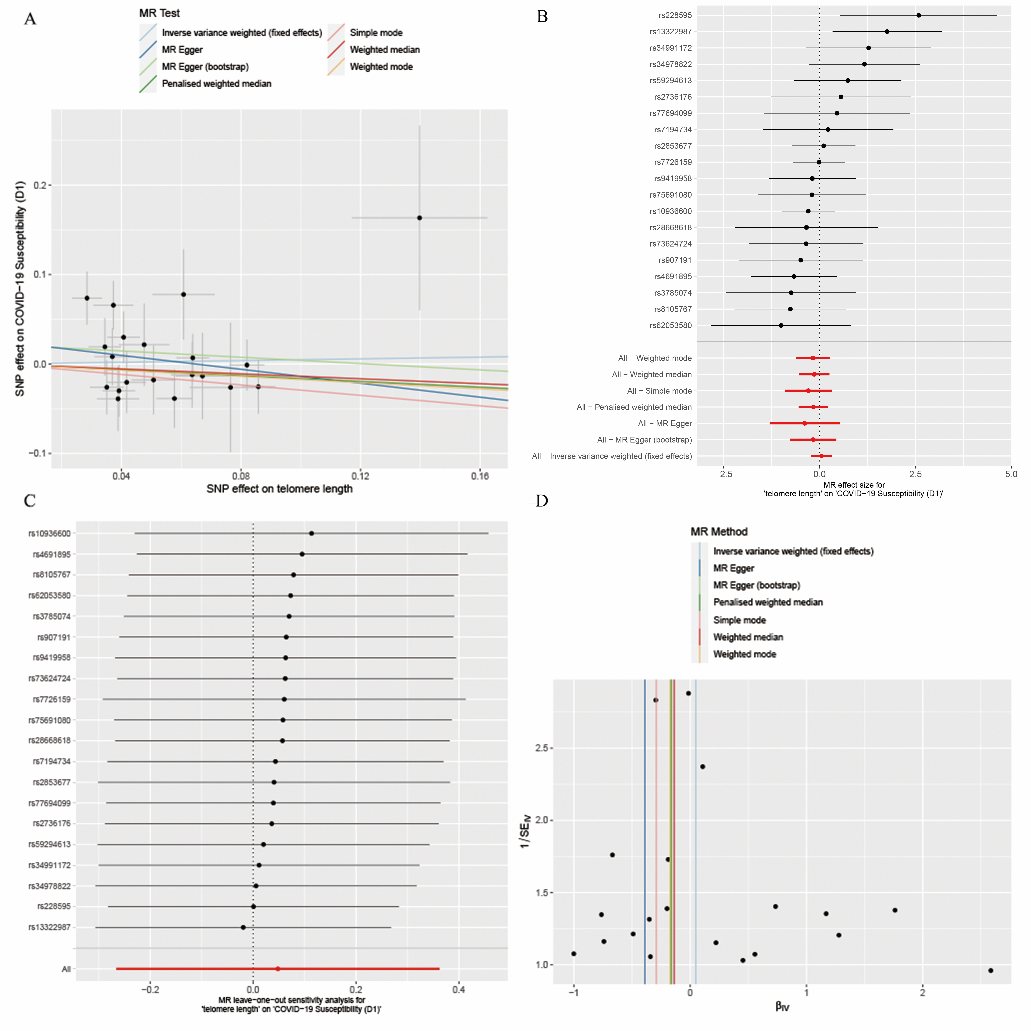


**Supplementary Fig. 4 MR Plots for Relationship of Genetically Predicted LTL with COVID-19 Susceptibility (D1).**

**A.** Scatterplot of SNP potential effects on LTL vs COVID-19 Susceptibility (D1), with the slope of each line corresponding to estimated MR effect per method. **B.** Forest plot of individual and combined SNP MR-estimated effect sizes based on seven methods. Data are expressed as raw OR values with 95% CI in 20 SNP set. **C.** Leave-one-out plots for heterogeneous MR LTL-COVID-19 Susceptibility (D1) associations. **D.** Funnel plot of the LTL instrument precision versus the MR effect estimates on the COVID-19 Susceptibility (D1).

**
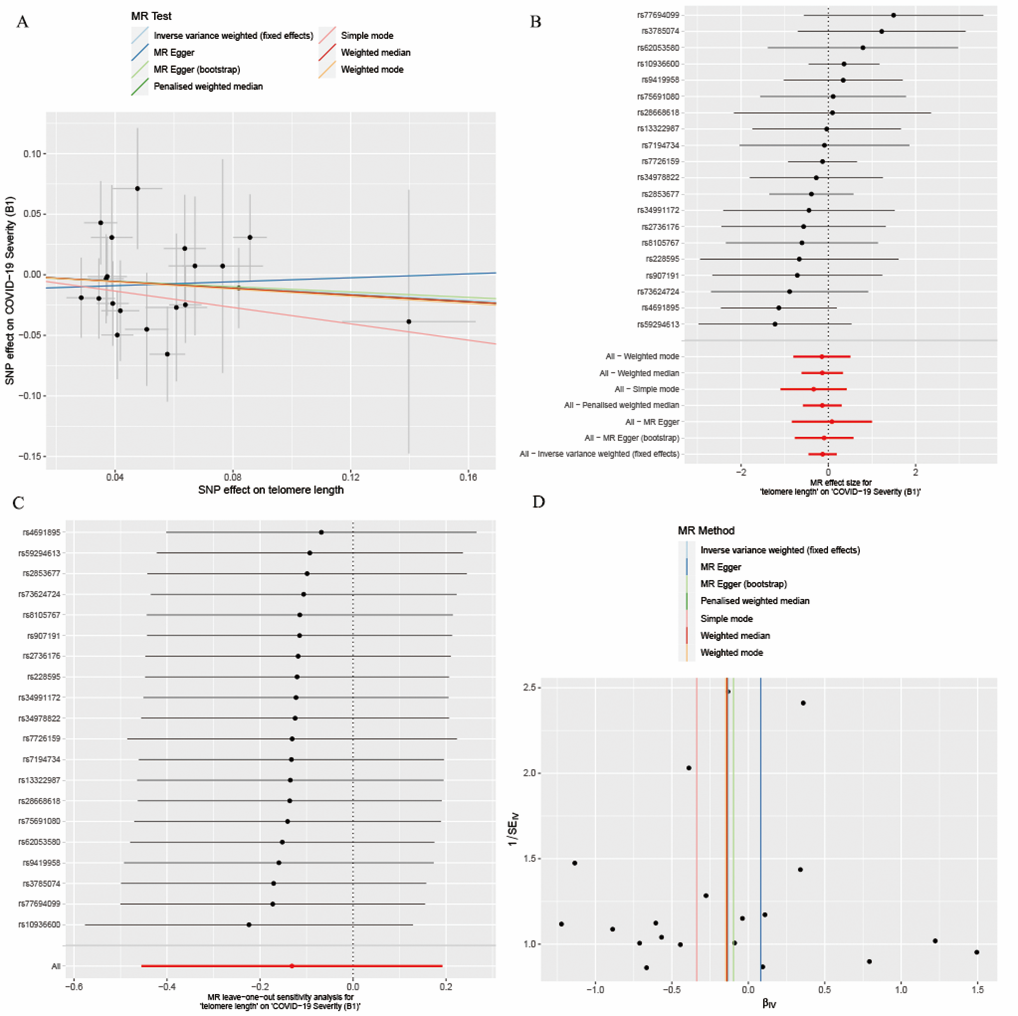
**

**Supplementary Fig. 5 MR Plots for Relationship of Genetically Predicted LTL with** **COVID-19 Severity (B1).**

**A.** Scatterplot of SNP potential effects on LTL vs COVID-19 Severity (B1), with the slope of each line corresponding to estimated MR effect per method. **B.** Forest plot of individual and combined SNP MR-estimated effect sizes based on seven methods. Data are expressed as raw OR values with 95% CI in 20 SNP set. **C.** Leave-one-out plots for heterogeneous MR LTL-COVID-19 Severity (B1) associations. **D.** Funnel plot of the LTL instrument precision versus the MR effect estimates on the COVID-19 Severity (B1).

**
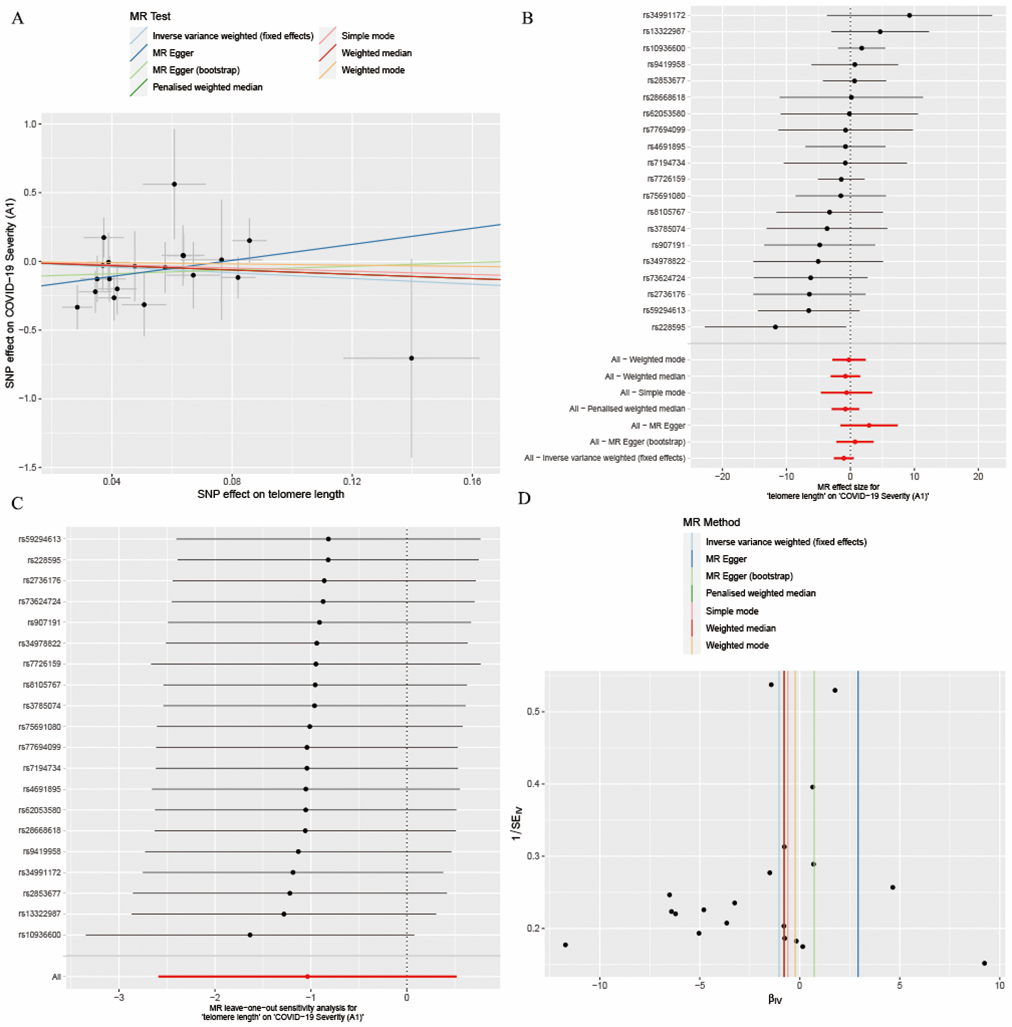
**

**Supplementary Fig. 6 MR Plots for Relationship of Genetically Predicted LTL with COVID-19 Severity (A1).**

**A.** Scatterplot of SNP potential effects on LTL vs COVID-19 Severity (A1), with the slope of each line corresponding to estimated MR effect per method. **B.** Forest plot of individual and combined SNP MR-estimated effect sizes based on seven methods. Data are expressed as raw OR values with 95% CI in 20 SNP set. **C.** Leave-one-out plots for heterogeneous MR LTL-COVID-19 Severity (A1) associations. **D.** Funnel plot of the LTL instrument precision versus the MR effect estimates on the COVID-19 Severity (A1).

**
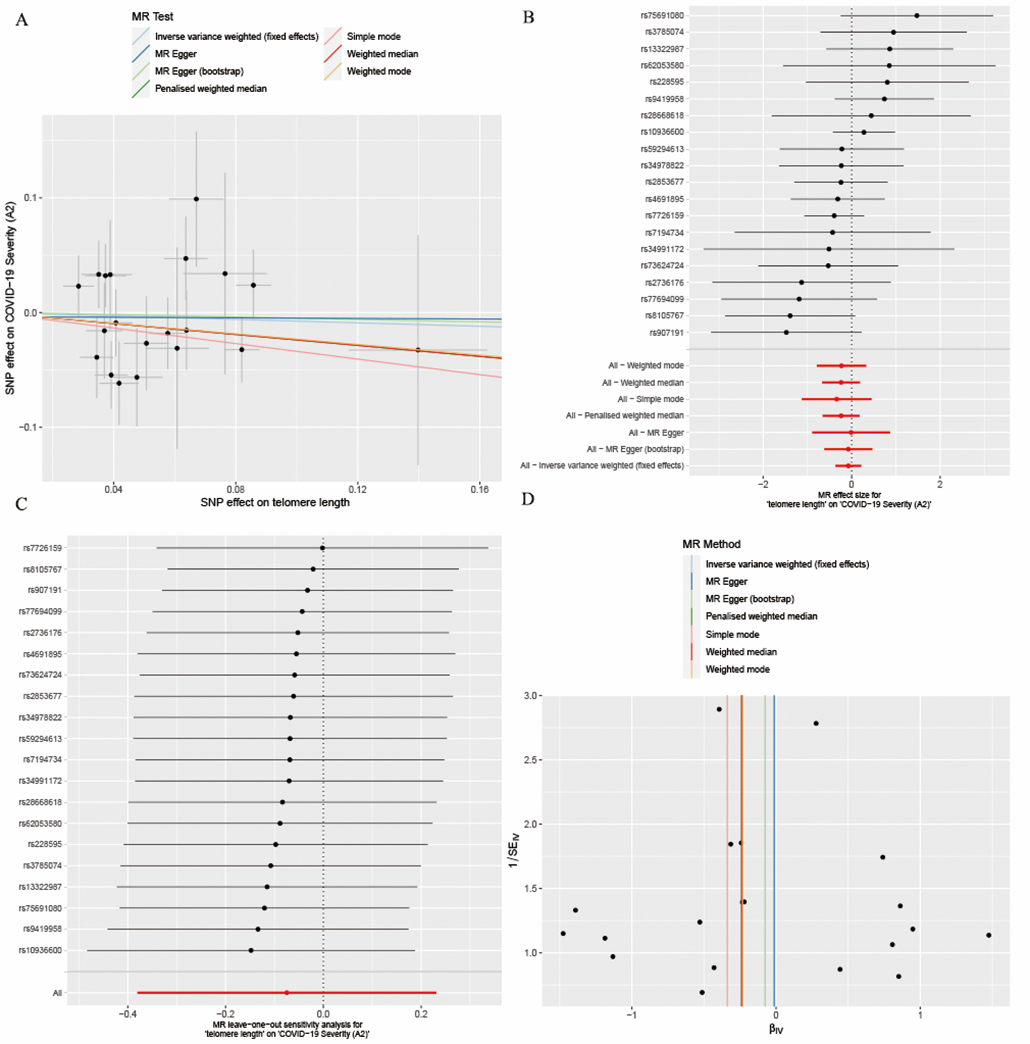
**

**Supplementary Fig. 7 MR Plots for Relationship of Genetically Predicted LTL with COVID-19 Severity (A2).**

**A.** Scatterplot of SNP potential effects on LTL vs COVID-19 Severity (A2), with the slope of each line corresponding to estimated MR effect per method. **B.** Forest plot of individual and combined SNP MR-estimated effect sizes based on seven methods. Data are expressed as raw OR values with 95% CI in 20 SNP set. **C.** Leave-one-out plots for heterogeneous MR LTL-COVID-19 Severity (A2) associations. **D.** Funnel plot of the LTL instrument precision versus the MR effect estimates on the COVID-19 Severity (A2).

**
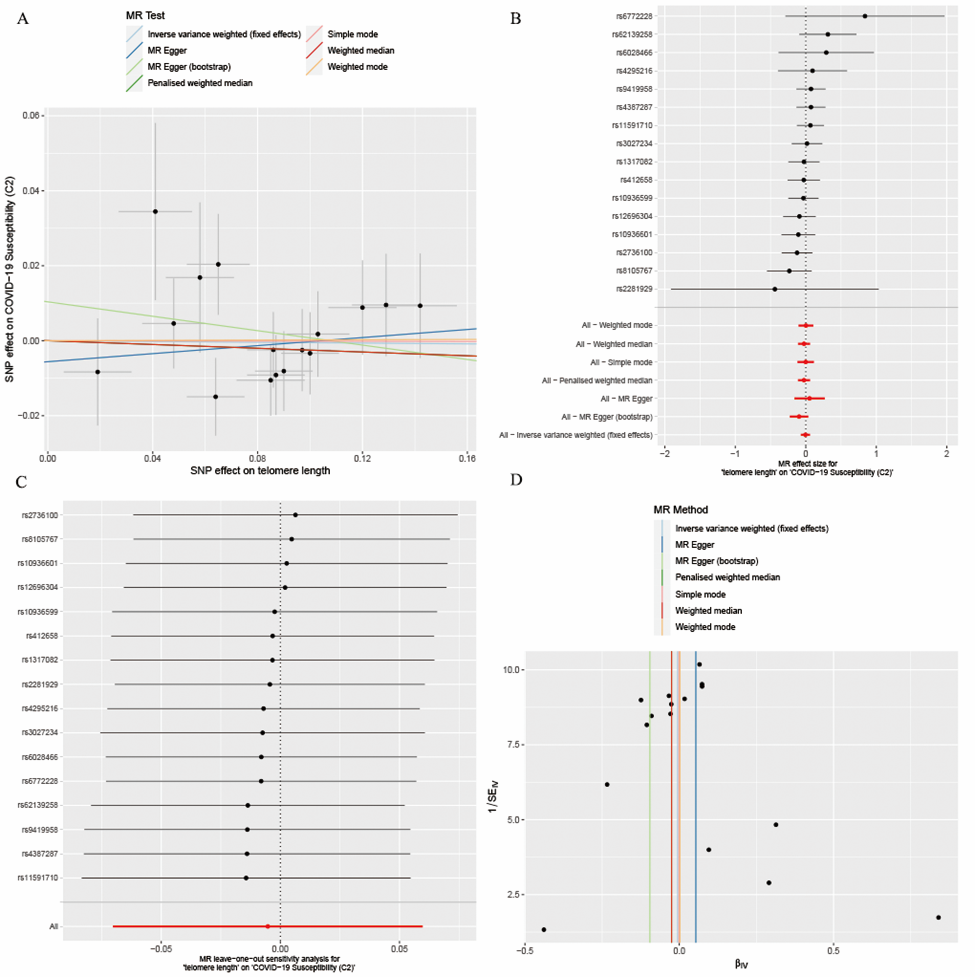
**

**Supplementary Fig. 8 MR Plots for Relationship of Genetically Predicted LTL with COVID-19 Susceptibility (C2).**

**A.** Scatterplot of SNP potential effects on LTL vs COVID-19 Susceptibility (C2), with the slope of each line corresponding to estimated MR effect per method. **B.** Forest plot of individual and combined SNP MR-estimated effect sizes based on seven methods. Data are expressed as raw OR values with 95% CI in 16 SNP set. **C.** Leave-one-out plots for heterogeneous MR LTL-COVID-19 Susceptibility (C2) associations. **D.** Funnel plot of the LTL instrument precision versus the MR effect estimates on the COVID-19 Susceptibility (C2).


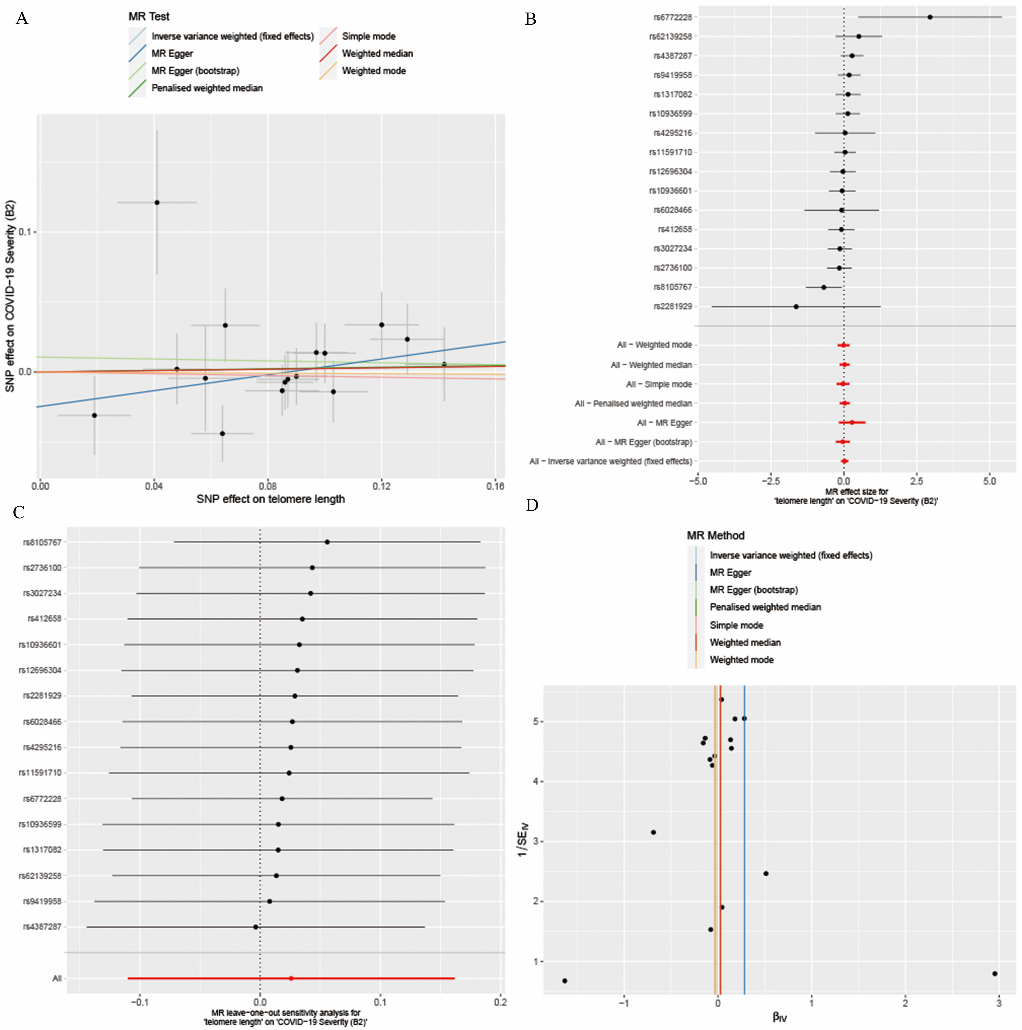


**Supplementary Fig. 9 MR Plots for Relationship of Genetically Predicted LTL with COVID-19 Severity (B2).**

**A.** Scatterplot of SNP potential effects on LTL vs COVID-19 Severity (B2), with the slope of each line corresponding to estimated MR effect per method. **B.** Forest plot of individual and combined SNP MR-estimated effect sizes based on seven methods. Data are expressed as raw OR values with 95% CI in 16 SNP set. **C.** Leave-one-out plots for heterogeneous MR LTL-COVID-19 Severity (B2) associations. **D.** Funnel plot of the LTL instrument precision versus the MR effect estimates on the COVID-19 Severity (B2).


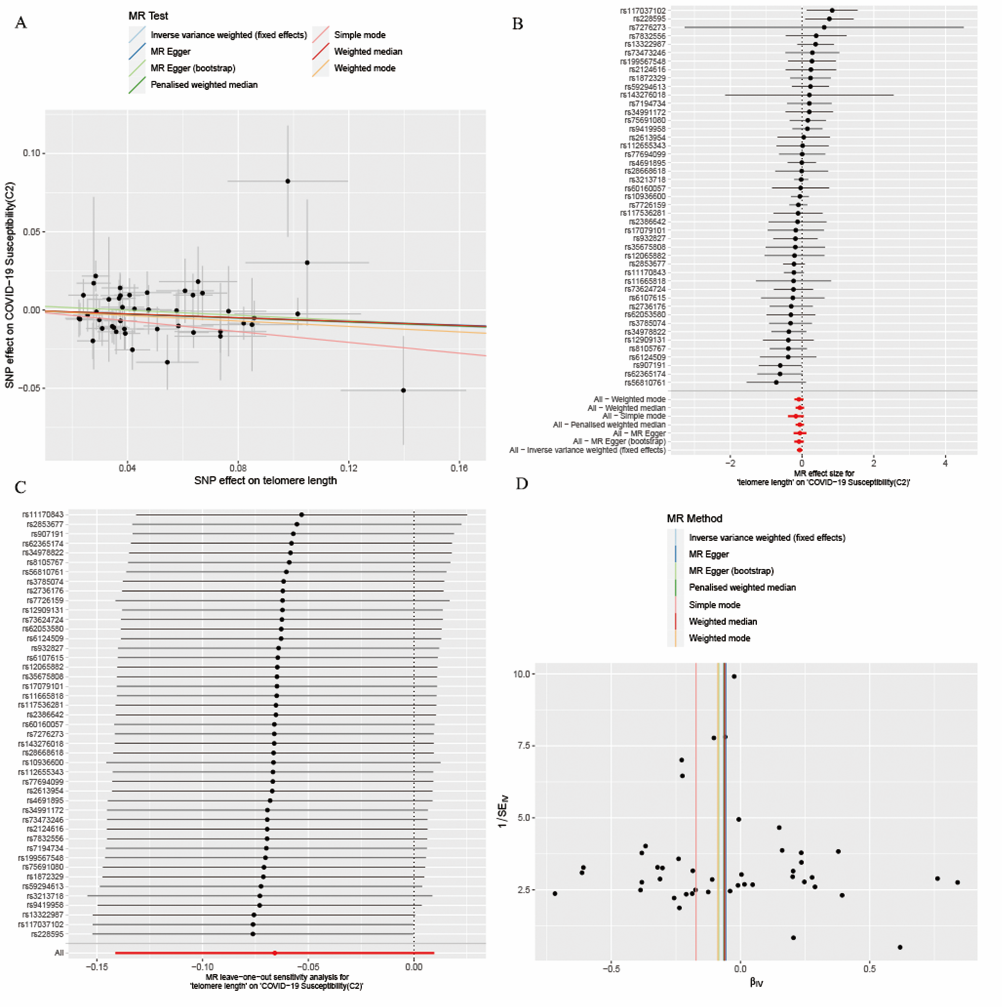


**Supplementary Fig. 10 MR Plots for Relationship of Genetically Predicted LTL with COVID-19 Susceptibility (C2).**

**A.** Scatterplot of SNP potential effects on LTL vs COVID-19 Susceptibility (C2), with the slope of each line corresponding to estimated MR effect per method. **B.** Forest plot of individual and combined SNP MR-estimated effect sizes based on seven methods. Data are expressed as raw OR values with 95% CI in 52 SNP set. **C.** Leave-one-out plots for heterogeneous MR LTL-COVID-19 Susceptibility (C2) associations. **D.** Funnel plot of the LTL instrument precision versus the MR effect estimates on the COVID-19 Susceptibility (C2).

**
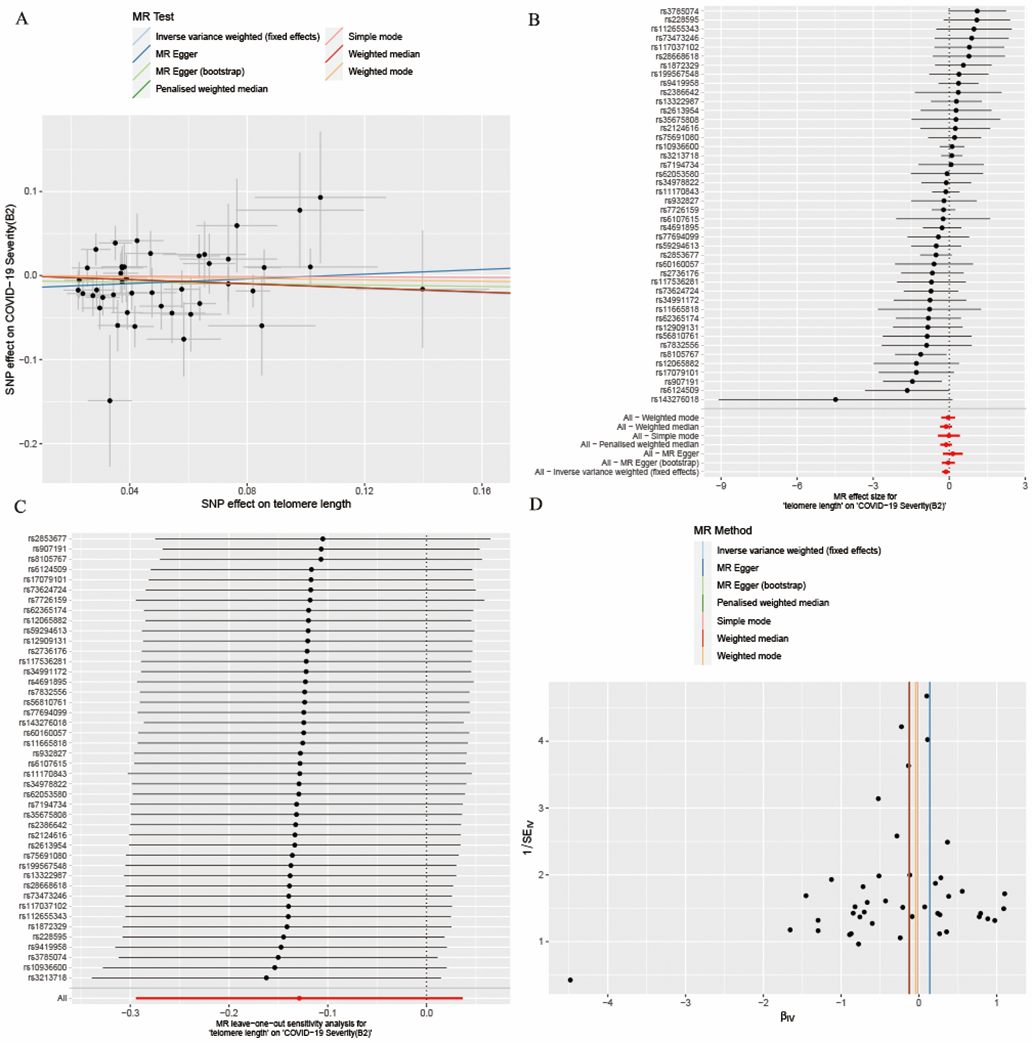
**

**Supplementary Fig. 11 MR Plots for Relationship of Genetically Predicted LTL with COVID-19 Severity (B2).**

**A.** Scatterplot of SNP potential effects on LTL vs COVID-19 Severity (B2), with the slope of each line corresponding to estimated MR effect per method. **B.** Forest plot of individual and combined SNP MR-estimated effect sizes based on seven methods. Data are expressed as raw OR values with 95% CI in 52 SNP set. **C.** Leave-one-out plots for heterogeneous MR LTL-COVID-19 Severity (B2) associations. **D.** Funnel plot of the LTL instrument precision versus the MR effect estimates on the COVID-19 Severity (B2).

**Supplementary Table 14 Results of reverse MR analyses**

| **Exposure** | **Outcome** | **Method** | **Beta** | **95% CI** | **SE** | **P value^*^** | **Heterogeneity P** | **Intercept**  **P** | **I^2^(%)** | **MR-Presso**  **P** |
| --- | --- | --- | --- | --- | --- | --- | --- | --- | --- | --- |
| Susceptibility(C2) | LTL | Inverse variance weighted | -0.022 | -0.11 to 0.07 | 0.046 | 0.630 | 0.822 | NA | NA | 0.776 |
|  |  | MR Egger | -0.053 | -0.32 to 0.21 | 0.134 | 0.721 | 0.690 | 0.824 | 98.99 |  |
|  |  | MR Egger (bootstrap) | -0.107 | -0.27 to 0.06 | 0.084 | 0.103 | NA | NA | NA |  |
|  |  | Penalised weighted median | -0.029 | -0.14 to 0.08 | 0.056 | 0.605 | NA | NA | NA |  |
|  |  | Simple mode | -0.002 | -0.14 to 0.14 | 0.069 | 0.978 | NA | NA | NA |  |
|  |  | Weighted median | -0.029 | -0.14 to 0.08 | 0.055 | 0.600 | NA | NA | NA |  |
|  |  | Weighted mode | -0.032 | -0.15 to 0.09 | 0.060 | 0.627 | NA | NA | NA |  |
| Severity(B2) | LTL | Inverse variance weighted | -0.001 | -0.04 to 0.04 | 0.022 | 0.949 | 0.826 | NA | NA | 0.822 |
|  |  | MR Egger | 0.020 | -0.06 to 0.10 | 0.040 | 0.636 | 0.784 | 0.549 | 99.03 |  |
|  |  | MR Egger (bootstrap) | -0.023 | -0.11 to 0.06 | 0.043 | 0.294 | NA | NA | NA |  |
|  |  | Penalised weighted median | 0.008 | -0.04 to 0.06 | 0.025 | 0.741 | NA | NA | NA |  |
|  |  | Simple mode | 0.010 | -0.07 to 0.09 | 0.039 | 0.810 | NA | NA | NA |  |
|  |  | Weighted median | 0.008 | -0.04 to 0.05 | 0.024 | 0.729 | NA | NA | NA |  |
|  |  | Weighted mode | 0.008 | -0.05 to 0.06 | 0.027 | 0.785 | NA | NA | NA |  |
| Severity(A2) | LTL | Inverse variance weighted | 0.009 | -0.02 to 0.04 | 0.017 | 0.593 | 0.905 | NA | NA | 0.942 |
|  |  | MR Egger | 0.015 | -0.05 to 0.08 | 0.035 | 0.689 | 0.804 | 0.852 | 98.66 |  |
|  |  | MR Egger (bootstrap) | 0.000 | -0.07 to 0.07 | 0.037 | 0.499 | NA | NA | NA |  |
|  |  | Penalised weighted median | 0.008 | -0.03 to 0.05 | 0.020 | 0.697 | NA | NA | NA |  |
|  |  | Simple mode | 0.021 | -0.03 to 0.07 | 0.027 | 0.475 | NA | NA | NA |  |
|  |  | Weighted median | 0.008 | -0.03 to 0.05 | 0.020 | 0.699 | NA | NA | NA |  |
|  |  | Weighted mode | 0.009 | -0.03 to 0.05 | 0.021 | 0.696 | NA | NA | NA |  |

*P values shown have not been corrected/adjusted for multiple comparisons

**
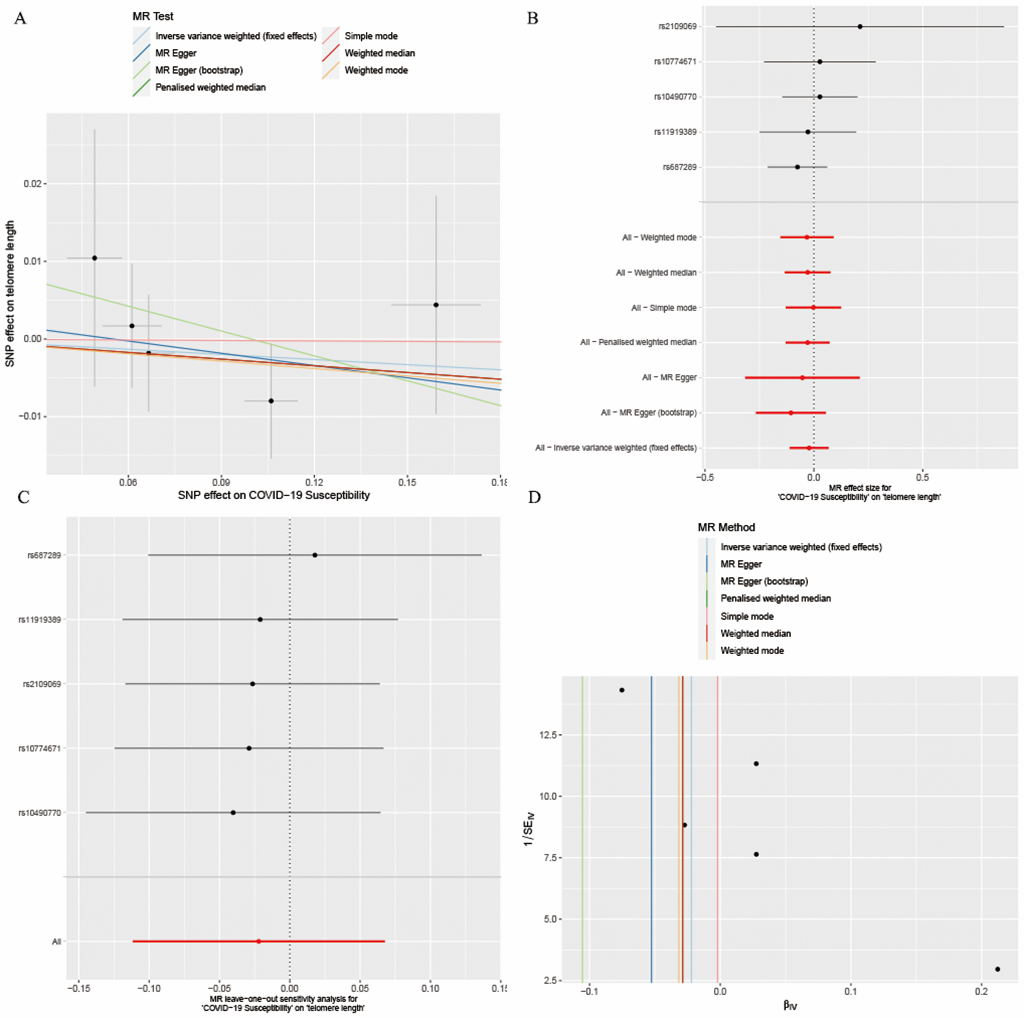
**

**Supplementary Fig. 12 Reverse MR Plots for Relationship of Genetically Predicted COVID-19 Susceptibility (C2) with LTL.**

**A.** Scatterplot of SNP potential effects on COVID-19 Susceptibility (C2) vs LTL, with the slope of each line corresponding to estimated MR effect per method. **B.** Forest plot of individual and combined SNP MR-estimated effect sizes based on seven methods. Data are expressed as raw Beta values with 95% CI in 5 SNP set. **C.** Leave-one-out plots for heterogeneous MR COVID-19 Susceptibility (C2)-LTL associations. **D.** Funnel plot of the COVID-19 Susceptibility (C2) instrument precision versus the MR effect estimates on the LTL.


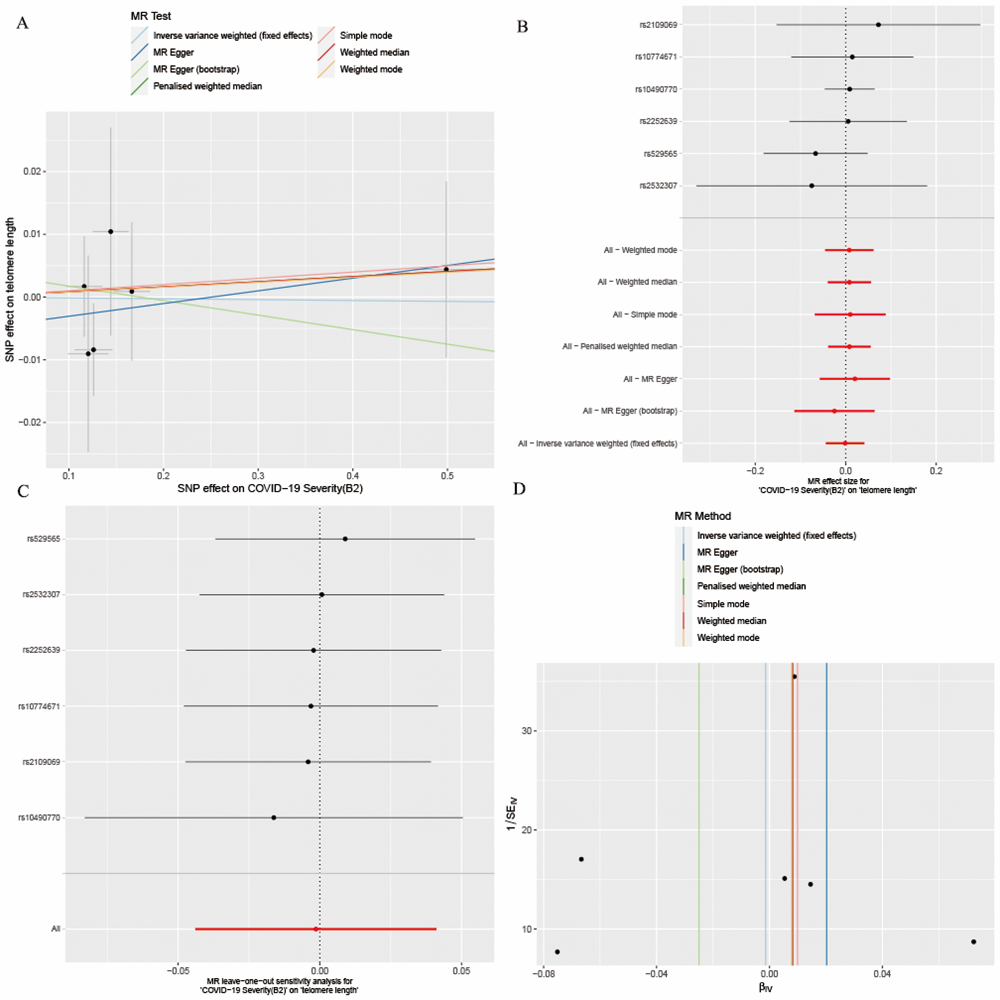


**Supplementary Fig. 13 Reverse MR Plots for Relationship of Genetically Predicted COVID-19 Severity (B2) with LTL.**

**A.** Scatterplot of SNP potential effects on COVID-19 Severity (B2) vs LTL, with the slope of each line corresponding to estimated MR effect per method. **B.** Forest plot of individual and combined SNP MR-estimated effect sizes based on seven methods. Data are expressed as raw Beta values with 95% CI in 6 SNP set. **C.** Leave-one-out plots for heterogeneous MR COVID-19 Severity (B2)-LTL associations. **D.** Funnel plot of the COVID-19 Severity (B2) instrument precision versus the MR effect estimates on the LTL.


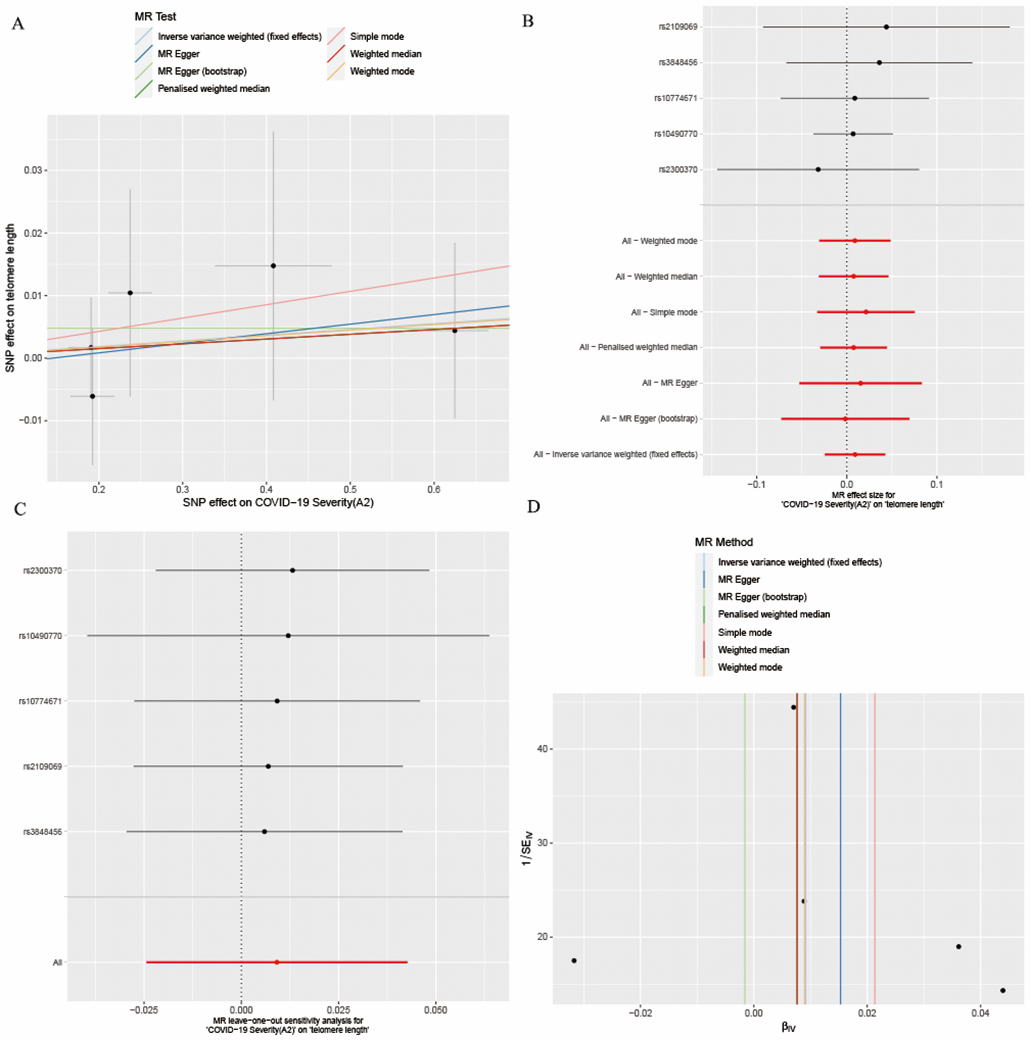


**Supplementary Fig. 14 Reverse MR Plots for Relationship of Genetically Predicted COVID-19 Severity (A2) with LTL.**

**A.** Scatterplot of SNP potential effects on COVID-19 Severity (A2) vs LTL, with the slope of each line corresponding to estimated MR effect per method. **B.** Forest plot of individual and combined SNP MR-estimated effect sizes based on seven methods. Data are expressed as raw Beta values with 95% CI in 5 SNP set. **C.** Leave-one-out plots for heterogeneous MR COVID-19 Severity (A2) -LTL associations. **D.** Funnel plot of the COVID-19 Severity (A2) instrument precision versus the MR effect estimates on the LTL.

| Supplementary Table 15 Genetic Correlation Estimates from LDSC Regression | | | | |
| --- | --- | --- | --- | --- |
| Phenotype 1 | Phenotype 2 | r_g_(SE) | P | r_g_ intercept(SE) |
| LTL | COVID-19 Susceptibility(C2) | -0.0532(0.1359) | 0.696 | 0.0024(0.0051) |
| LTL | COVID-19 Severity (B2) | -0.1123(0.118) | 0.341 | 0.0004(0.0049) |
| LTL | COVID-19 Severity (A2) | 0.06(0.1199) | 0.617 | -0.004(0.005) |

| Supplementary Table 16 Power calculations for MR analysis of the effect of genetically predicted LTL on COVID-19 phenotypes | | | | | | | |
| --- | --- | --- | --- | --- | --- | --- | --- |
| Fraction of variance in LTL explained by 20 SNPs (1.773%) | | | | | | | |
| COVID-19  Phenotype | OR=0.7 | OR=0.75 | OR=0.8 | OR=0.85 | OR=0.9 | Sample size for outcome | Proportion of cases |
| C2 | 100% | 100% | 100% | 98.8% | 78.2% | 1,683,768 | 0.023 |
| B2 | 99.7% | 96.8% | 84.2% | 57.8% | 28.7% | 1,887,658 | 0.0053 |
| Fraction of variance in LTL explained by 52 SNPs (2.647%) | | | | | | | |
| COVID-19  Phenotype | OR=0.7 | OR=0.75 | OR=0.8 | OR=0.85 | OR=0.9 | Sample size for outcome | Proportion of cases |
| C2 | 100% | 100% | 100% | 99.9% | 91.7% | 1,683,768 | 0.023 |
| B2 | 100% | 99.7% | 95.1% | 75.0% | 40.1% | 1,887,658 | 0.0053 |
| Fraction of variance in LTL explained by 16 SNPs (3.566%) | | | | | | | |
| COVID-19  Phenotype | OR=0.7 | OR=0.75 | OR=0.8 | OR=0.85 | OR=0.9 | Sample size for outcome | Proportion of cases |
| C2 | 100% | 100% | 100% | 100% | 97.3% | 1,683,768 | 0.023 |
| B2 | 100% | 100% | 98.7% | 86.4% | 50.9% | 1,887,658 | 0.0053 |

The power for our MR study were calculated using an online tool (https://sb452.shinyapps.io/power/)
